# Sklerosierende Erkrankungen der Haut

**DOI:** 10.1111/ddg.15835_g

**Published:** 2025-10-23

**Authors:** Yasamin Kalantari, Katharina Meier, Kamran Ghoreschi, Maria Kinberger, Farzan Solimani

**Affiliations:** ^1^ Klinik für Dermatologie Venerologie und Allergologie Charité – Universitätsmedizin Berlin; ^2^ Berlin Institute of Health an der Charité – Universitätsmedizin Berlin BIH Biomedical Innovation Academy BIH Charité Clinician Scientist Program, Berlin

**Keywords:** Fibrose, Morphea, Systemische sklerose, morphea, skin fibrosis, Systemic sclerosis

## Abstract

Sklerosierende Hautkrankheiten sind eine Gruppe unterschiedlicher dermatologischer Erkrankungen, die durch fibrotische Veränderungen gekennzeichnet sind und die Lebensqualität der Patienten stark einschränken können. Diese Erkrankungen treten häufig mit kutanen Manifestationen auf und können in bestimmten Fällen auch extrakutanes Gewebe betreffen, was zu erheblicher Morbidität und Mortalität führen kann. In dieser Übersichtsarbeit wird der aktuelle Kenntnisstand zu den vier häufigsten sklerosierenden Erkrankungen dargestellt: der lokalisierten Sklerodermie (Morphea), der systemischen Sklerose (SSc), dem Skleromyxödem und dem Scleroedema adultorum Buschke sowie sklerotischen Erkrankungen, die durch äußere Einflüsse verursacht werden. Hier werden die Pathophysiologie, die klinischen Merkmale und der Krankheitsverlauf dieser Entitäten erörtert. Darüber hinaus werden die Diagnosemethoden und Behandlungsmöglichkeiten im Detail beschrieben.

## EINLEITUNG

Sklerosierende Erkrankungen der Haut umfassen verschiedene dermatologische Erkrankungen, die durch fibrotische Veränderungen gekennzeichnet sind. Diese Erkrankungen reichen von lokalisierten Formen wie Morphea, die in erster Linie die Haut und das subkutane Gewebe betreffen, bis hin zur systemischen Sklerose (SSc), einer komplexen und schweren Autoimmunerkrankung, die mehrere Organsysteme, einschließlich der Lunge, des Herzens und des Magen‐Darm‐Trakts, betreffen kann und zu einer erheblichen Morbidität und Mortalität führen kann.^1^, ^2^, ^3^, ^4^, ^5^

Diese Erkrankungen können die Lebensqualität der Patienten erheblich beeinträchtigen und stellen aufgrund ihrer klinischen Heterogenität eine diagnostische und therapeutische Herausforderung dar. In den vergangenen Jahren wurden Fortschritte im therapeutischen Management erzielt, und aktuelle Forschungsarbeiten fokussieren zunehmend auf zielgerichtete Behandlungsansätze mit dem Ziel, die Behandlungsergebnisse für die Patienten zu verbessern.^5^ Eine der jüngsten Konsenserklärungen zur Diagnostik und Therapie sklerosierender Hauterkrankungen, veröffentlicht von Knobler et al.,^1^ bietet Ärzten eine umfassende Übersicht über Erst‐ und Zweitlinienbehandlungen.

Diese Übersichtsarbeit erörtert den aktuellen Wissensstand zu den vier häufigsten sklerosierenden Erkrankungen: der lokalisierten Sklerodermie (Morphea), der systemischen Sklerose (SSc), dem Skleromyxödem und dem Scleroedema adultorum Buschke.

## LOKALISIERTE SKLERODERMIE (MORPHEA)

### Klinisches Erscheinungsbild und Subtypen

Die lokalisierte Sklerodermie (Morphea) ist eine chronisch‐entzündliche Autoimmunerkrankung des Bindegewebes, die durch gestörte Kollagensynthese mit konsekutiver Hautverdickung und ‐verhärtung charakterisiert ist.

Obwohl die Morphea in erster Linie die Haut betrifft, kann sie in manchen Fällen auch tiefer gelegene Gewebe wie das subkutane Fettgewebe, Faszien, Muskeln und sogar Knochen erfassen.

Obwohl die Morphea in erster Linie die Haut betrifft, kann sie in manchen Fällen auch tiefer gelegene Gewebe wie das subkutane Fettgewebe, Faszien, Muskeln und sogar Knochen erfassen.^1^, ^2^, ^3^, ^4^, ^5^ In solchen Fällen können Komplikationen wie Gelenkkontrakturen, eingeschränkte Beweglichkeit oder Wachstumsstörungen im Kindesalter – etwa eine Längendifferenz der Extremitäten – auftreten. Ein Übergang von Morphea in eine systemische Sklerose, der von Patienten häufig befürchtet wird, ist nicht möglich.

Die Morphea lässt sich anhand des klinischen Erscheinungsbilds und der betroffenen Strukturen in verschiedene Subtypen einteilen. Entsprechend der von Kreuter et al.^5^ veröffentlichten Leitlinie wird folgende Klassifikation angewendet (Tabelle 1).

**TABELLE 1:** Klinische Subtypen der Morphea.

Lokalisierter Typ	Plaque‐Morphea
	Morphea guttata
	Atrophodermie von Pasini und Pierini (superfizielle Morphea)
	Morphea profunda
Generalisierter Typ	Generalisierte Morphea
	Pansklerotische Morphea
Lineare Morphea	Lineare Morphea der Extremitäten
	Morphea *en coup de sabre*
	Parry‐Romberg‐Syndrom, progressive Hemiatrophie des Gesichts
	Eosinophile Fasziitis (Shulman Syndrom)
Gemischte Formen der Morphea	

### Limitierte Formen

#### Plaque‐Morphea (Abbildung 1)

Die Plaque‐Morphea ist der häufigste Subtyp der begrenzten Morphea, insbesondere bei Erwachsenen. Sie äußert sich in ovalen, erythematösen Läsionen, die typischerweise am Rumpf und in der Leistenregion auftreten. Aktive Herde sind durch einen violetten Randsaum gekennzeichnet, der auch als „lila Ring“ bezeichnet wird. Im Verlauf nimmt die zentrale Induration zu und entwickelt eine weißliche bis elfenbeinfarbene Tönung mit glänzender Oberfläche. Die Läsionen werden zunehmend atrophisch und neigen dazu, Haarfollikel sowie Hautanhangsgebilde zu schädigen, was zu postinflammatorischer Hyperpigmentierung führt.^4^, ^6^, ^7^

#### Morphea guttata (Abbildung 2b,c)

Die Morphea guttata manifestiert sich in Form multipler gelb‐weißer, münzförmiger Läsionen am Rumpf, die klein (< 1 cm) und glänzend sind. Frühstadien können sich auch als kleine erythematöse Makulae präsentieren.^8^

#### Atrophodermie von Pasini und Pierini (Abbildung 3)

Die Atrophodermie von Pasini und Pierini (oberflächliche Morphea) ist ein seltener Subtyp, der überwiegend im Kindesalter auftritt.^5^ Sie ist durch unterschiedlich große, symmetrische, rund bis oval konfigurierte, erythematöse oder graubraune Hautvertiefungen mit steil abfallenden Rändern gekennzeichnet. Die Einsenkungen entstehen durch einen Verlust von Bindegewebe.^8^, ^9^

#### Tiefe Morphea (Abbildung 2a)

Die tiefe Morphea ist der seltenste begrenzte Subtyp der Morphea und tritt bei weniger als 1  % der Patienten auf.^5^ Sie betrifft tiefere Bindegewebsschichten wie subkutanes Fettgewebe, Faszien und Muskulatur und manifestiert sich typischerweise in Form schmerzhafter Läsionen an den Extremitäten.^8^

### Generalisierte Formen

#### Generalisierte Morphea (Abbildung 4)

Die generalisierte Morphea ist eine schwerere und ausgedehntere Verlaufsform, die bei etwa 7  % bis 9  % der Patienten auftritt.^6^ Sie ist definiert durch die Beteiligung von mindestens drei anatomischen Regionen – typischerweise Rumpf, lumbosakraler Bereich und Oberschenkel.^5^ Die Läsionen sind in der Regel symmetrisch angeordnet und neigen dazu, zu größeren Plaques zu konfluieren.

#### Pansklerotische Morphea

Diese stark beeinträchtigende Verlaufsform der Morphea betrifft häufig Kinder unter 14 Jahren. Eine rasch fortschreitende Fibrose der tiefen Gewebe bis hin zur Pansklerose führt zu erheblicher Morbidität mit Gelenkkontrakturen, Längendifferenzen der Extremitäten und Immobilität sowie gestörter Wundheilung.^5^, ^10^

### Lineare Formen

#### Lineare Morphea der Extremitäten (Abbildung 5)

Bei dieser Form handelt es sich in der Regel um eine einzelne lineare und einseitige Läsion an den Extremitäten, die manchmal den Linien von Blaschko folgt. Während leichte Formen unter Umständen unter Zurücklassung einer Hyperpigmentierung abheilen, können schwerere Formen zu Beugekontrakturen und Bewegungseinschränkungen führen.^8^

#### Morphea en coup de sabre (Abbildung 6a,b)

Bei dieser Variante führt die lineare Morphea zu einer atrophischen Einsenkung an Kopf und Gesicht – meist in der frontoparietalen Region oder paramedianen Stirn, die an einen „Säbelhieb“ erinnert (*en coup de sabre*). Dieser Subtyp ist mit Komplikationen wie verschiedenen Formen der Alopezie und zerebraler Beteiligung assoziiert.^7^, ^8^

#### Parry‐Romberg‐Syndrom (Abbildung 6c)

Das Parry‐Romberg‐Syndrom, auch als progressive faziale Hemiatrophie bekannt, ist gekennzeichnet durch einseitige Atrophie des subkutanen Gewebes und der knöchernen Strukturen von Kopf und Gesicht, typischerweise ohne kutane Sklerose. Es kann zu neurologischer, okulärer und oraler Beteiligung kommen, und die Erkrankung führt häufig zu einer ausgeprägten fazialen Asymmetrie. Ein gleichzeitiges Auftreten mit der *En‐Coup‐de‐Sabre*‐Form ist häufig und wurde in bis zu 40  % der Fälle beschrieben.^7^, ^8^

#### Eosinophile Fasziitis

Die eosinophile Fasziitis, auch als Shulman‐Syndrom bekannt, manifestiert sich initial als akute Entzündungsreaktion in Form schmerzhafter, erythematöser Schwellungen und nicht eindrückbarer Ödeme der Extremitäten, wobei Finger und Zehen typischerweise ausgespart bleiben. Im weiteren Verlauf entwickelt sich eine tiefliegende Fibrose unter Einbeziehung der Faszien und subkutanen Septen, die zu einem charakteristischen *Peau‐d'Orange*‐Erscheinungsbild (Abbildung 7a) und venösen Einziehungen (*groove sign*, Abbildung 7b) führt. Bei betroffenen Patienten finden sich häufig eine periphere und gewebsbezogene Eosinophilie.

**ABBILDUNG 1:**
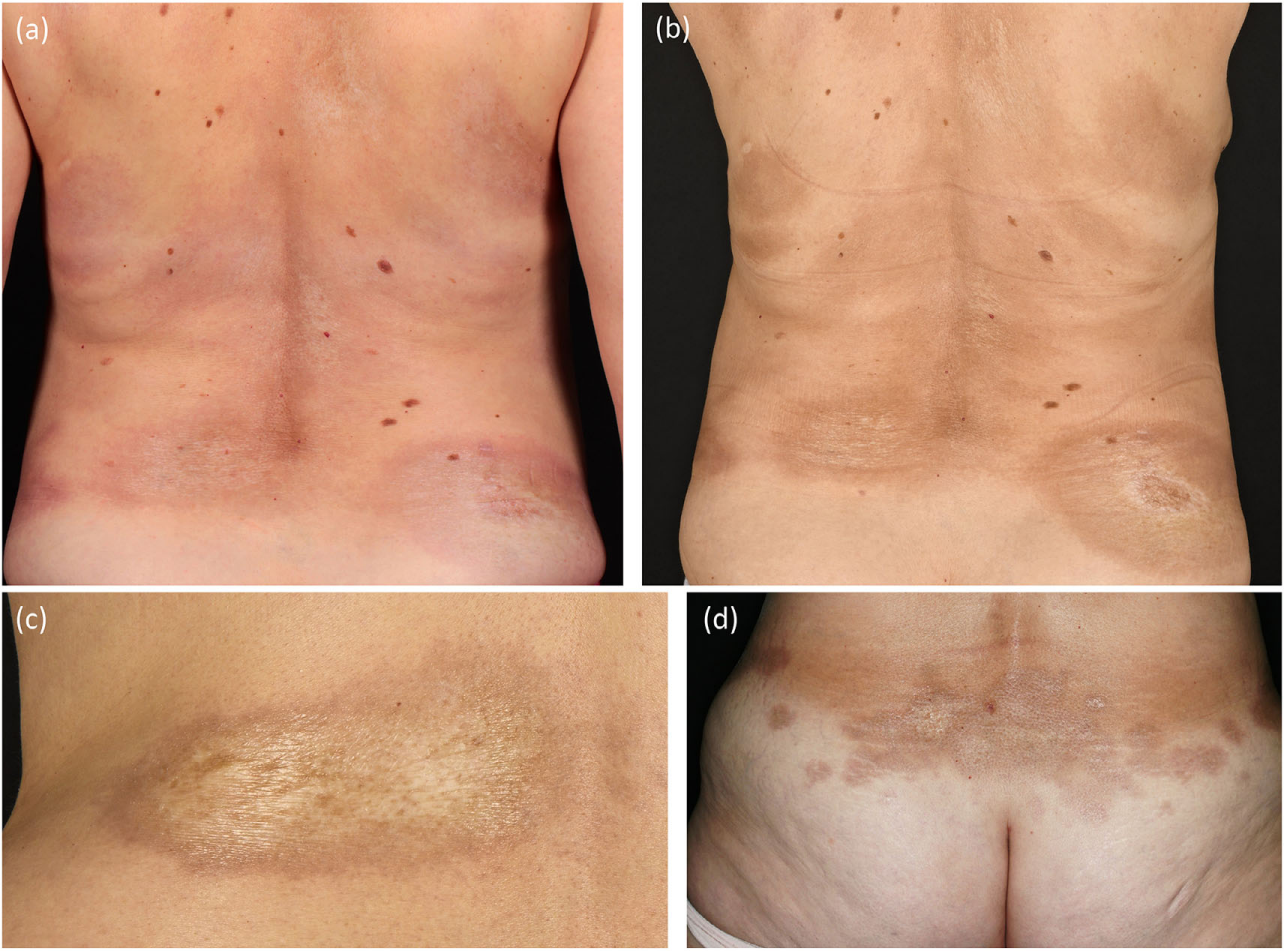
Klinische Heterogenität bei Plaque‐Typ‐Morphea. (a) Patientin mit aktiver Plaque‐Morphea mit lilafarbenen Ringen; (b) dieselbe Patientin nach Beginn der Behandlung; (c, d) sklerotische Plaques.

**ABBILDUNG 2:**
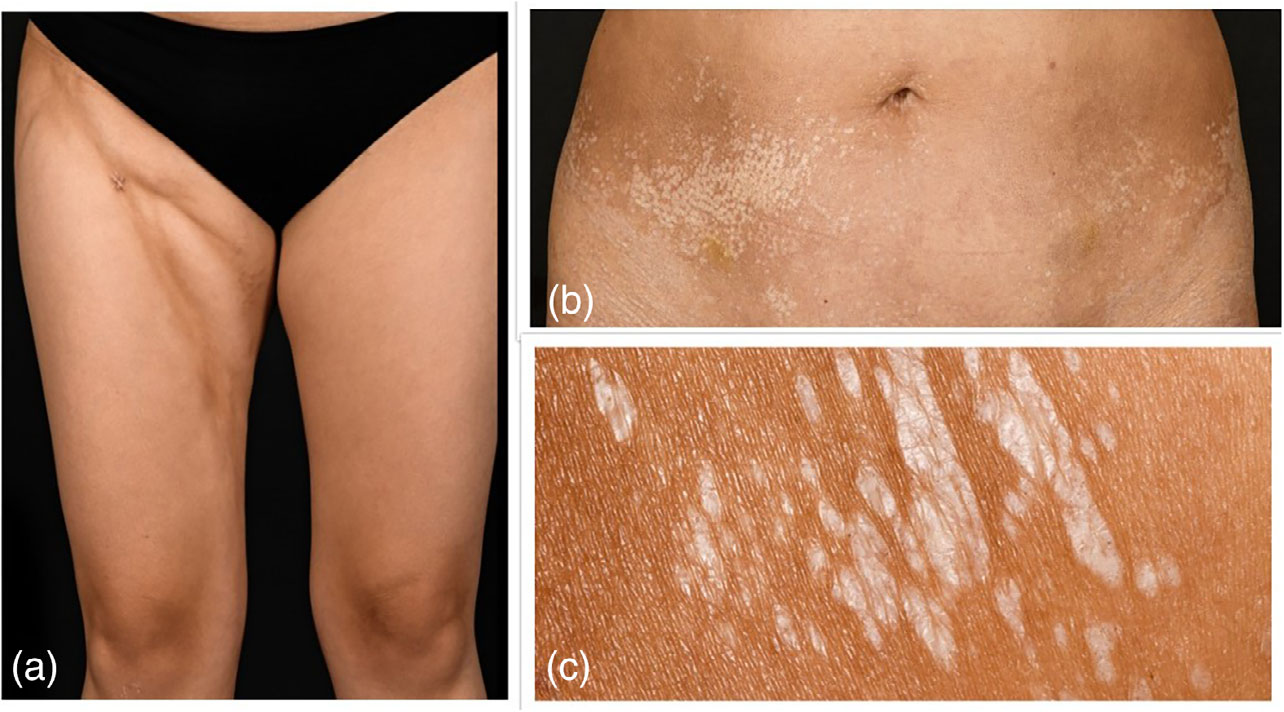
(a) Ein Patient mit tiefer Morphea; (b, c) ein Patient mit Morphea guttata.

**ABBILDUNG 3:**
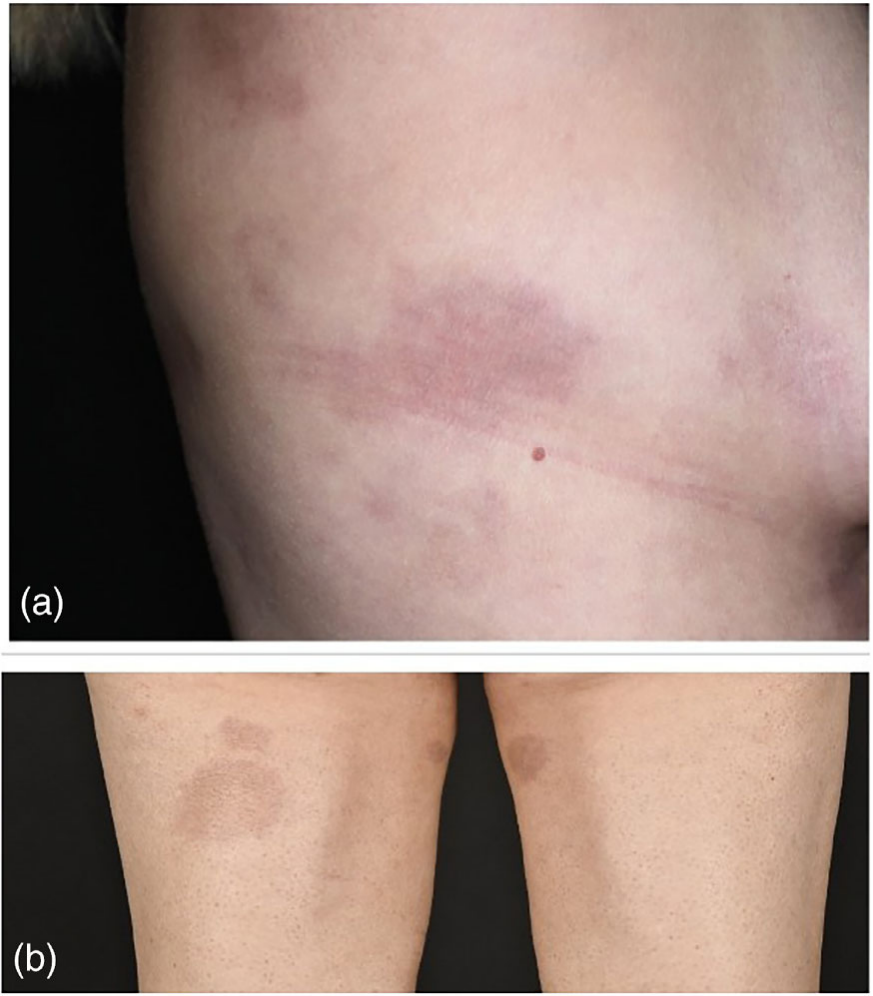
(a, b) Atrophodermie von Pasini und Pierini bei zwei verschiedenen Patienten.

**ABBILDUNG 4:**
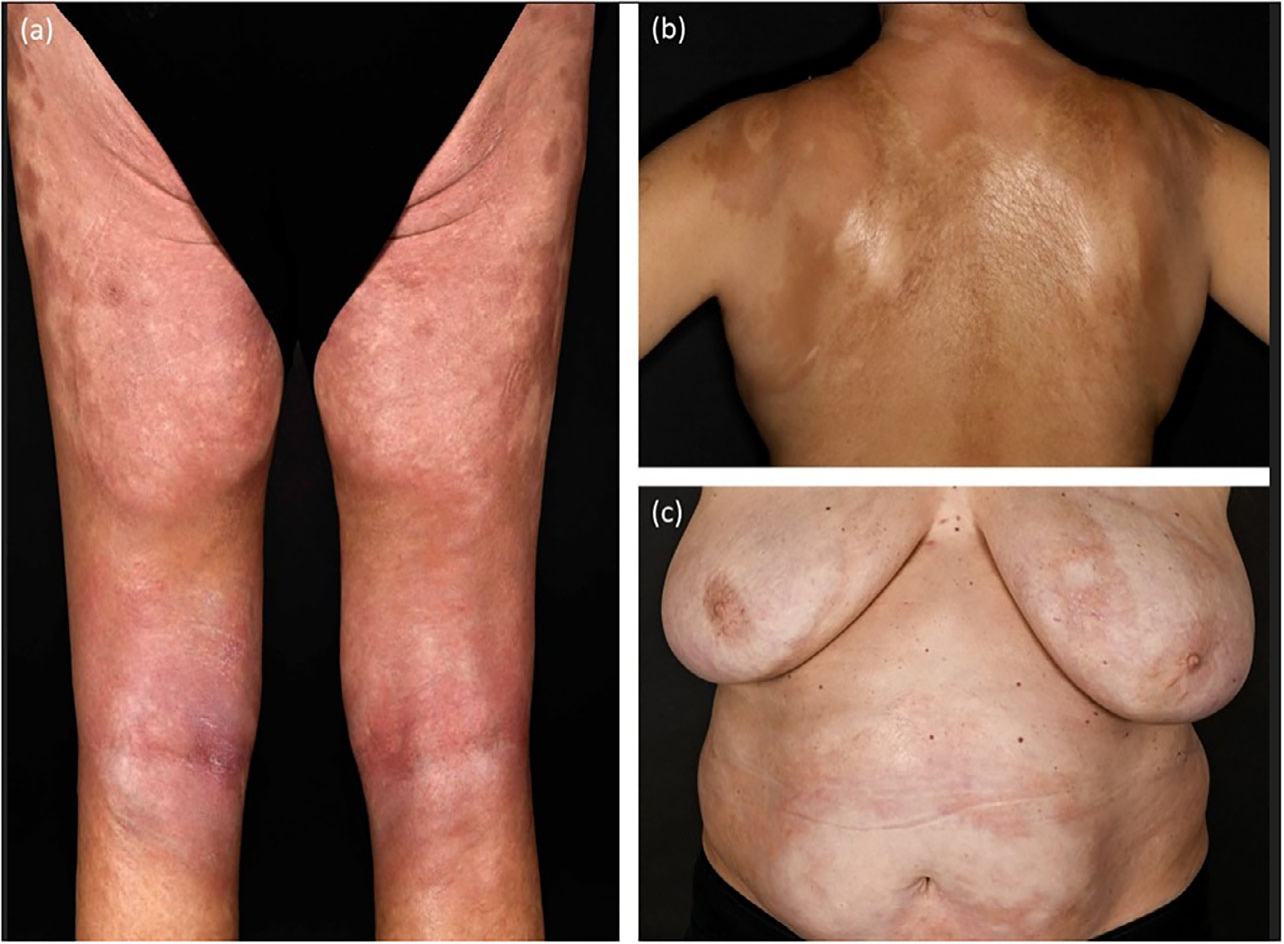
Klinische Heterogenität bei generalisierter Morphea. (a) Symmetrische, große sklerotische Plaques an den Beinen; (b) großflächige Sklerose am Rücken; (c) sklerotische Plaques am Oberkörper mit aktiven lilafarbenen Ringen.

**ABBILDUNG 5:**
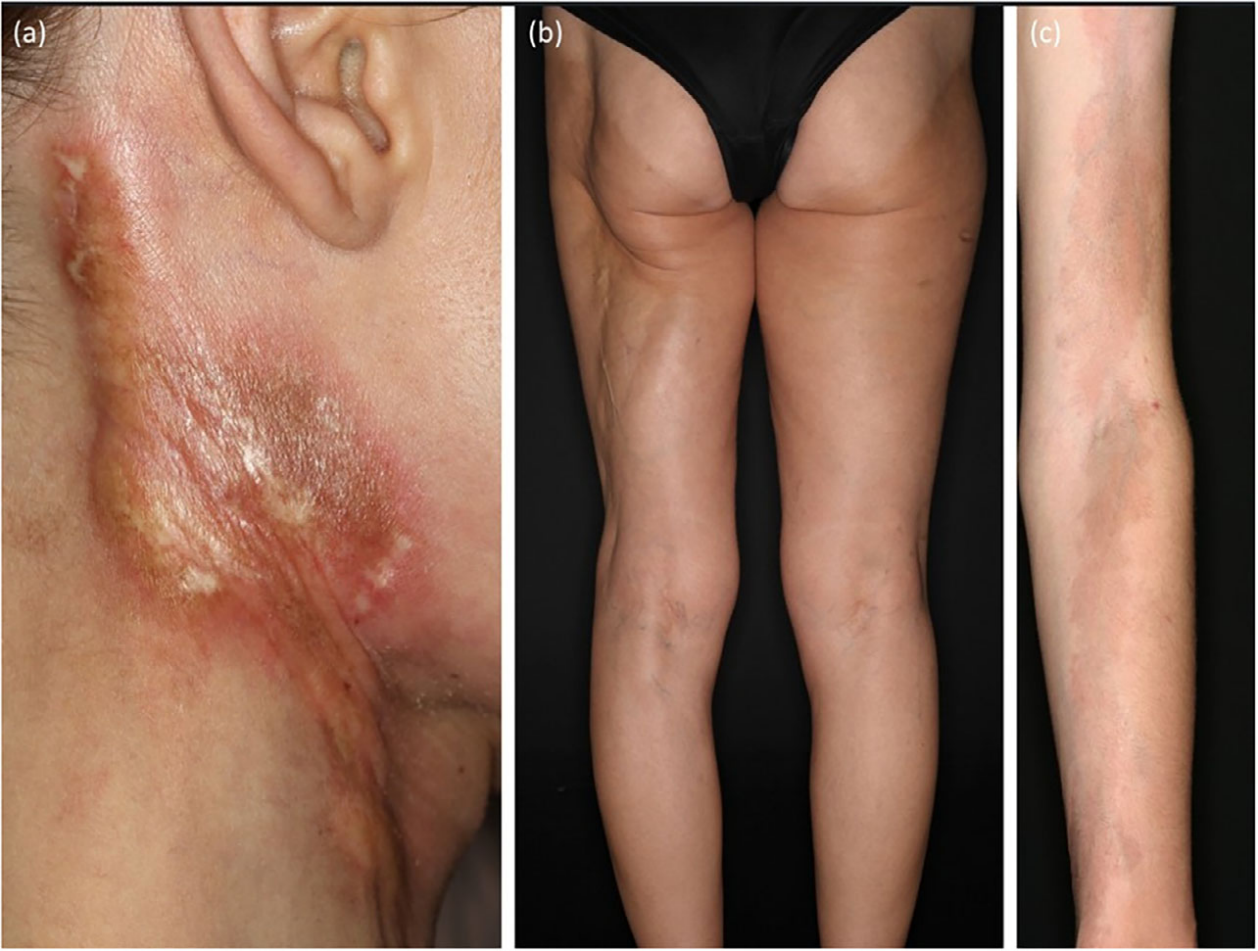
Klinische Heterogenität bei linearer Morphea. (a) Lineare Morphea am Hals mit sklerosiertem Zentrum und lilafarbenem Randsaum; (b) tiefe lineare Morphea am linken Bein; (c) oberflächliche lineare Morphea am Arm.

**ABBILDUNG 6:**
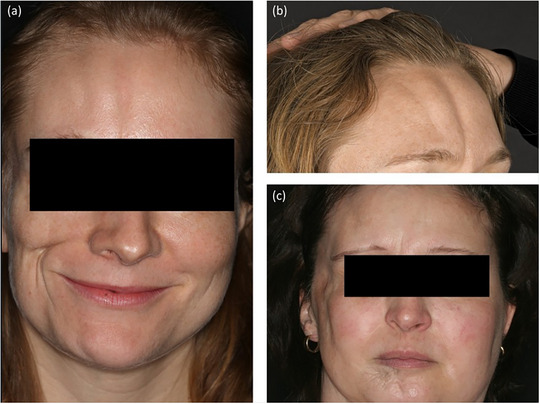
Tiefe Morphea im Gesicht. (a) Parry‐Romberg‐Syndrom mit Morphea *en coup de sabre*; (b) Morphea *en coup de sabre* mit zwei „Säbelstrichen“; (c) Parry‐Romberg‐Syndrom.

**ABBILDUNG 7:**
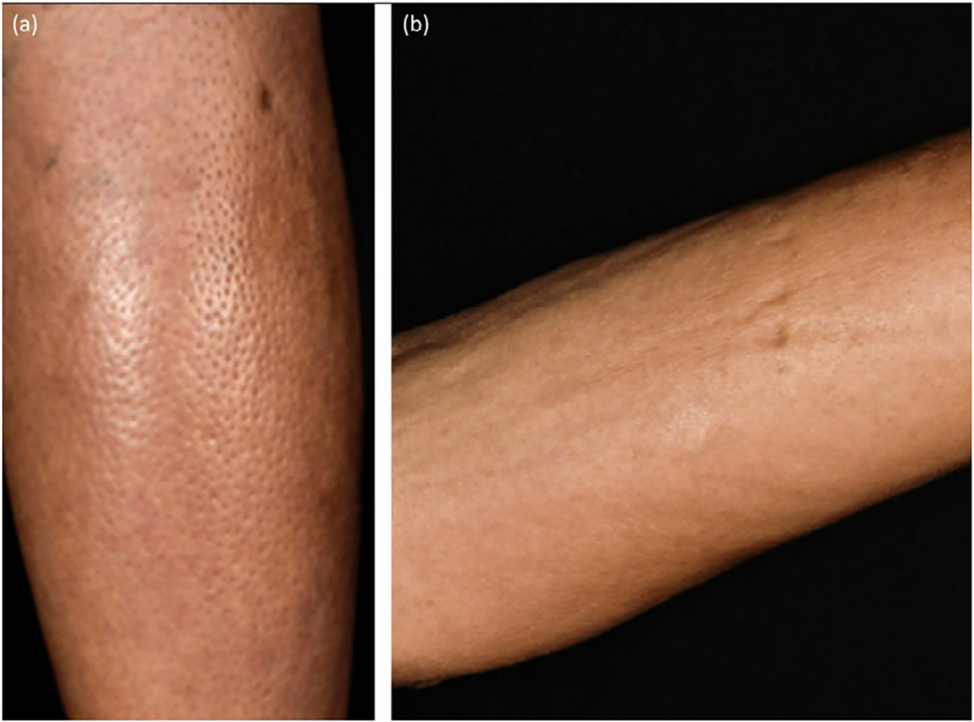
Eosinophile Fasziitis. (a) Orangenhaut‐Phänomen (*peau d'orange*); (b) Rillenzeichen.

#### Gemischte Morphea

Die Diagnose einer gemischten Morphea wird gestellt, wenn zwei oder mehr Formen der Morphea vorhanden sind. Die gemischte Morphea tritt bei 15 % der Patienten auf und ist häufig eine Kombination aus „linearem Typ und Plaque‐Morphea“ oder „linearem und generalisiertem Typ“.^7^

### Epidemiologie

Die weltweite Inzidenz der Morphea liegt bei 4 bis 27 Fällen pro Million Einwohner und betrifft Frauen 2,4‐ bis 4,2‐mal häufiger als Männer, insbesondere in ethnisch europäischen Bevölkerungsgruppen.^6^, ^7^ Bei Erwachsenen manifestiert sich die Erkrankung typischerweise in der fünften Lebensdekade, wobei die Plaque‐Morphea der am häufigsten beschriebene Subtyp ist. Bei Kindern liegt das Erkrankungsalter meist zwischen 2 und 14 Jahren; hier tritt vor allem die lineare Morphea auf.

### Ätiologie und Pathogenese

Die Pathogenese der Morphea ist bislang nicht vollständig geklärt und gilt als multifaktoriell. Bestimmte HLA‐Klasse‐I‐ (HLA‐B37) und Klasse‐II‐Allele (DRB104:04) sind mit der Erkrankung assoziiert. Morphea‐assoziierte Allele zeigen Überschneidungen mit rheumatoider Arthritis (RA), autoimmuner Schilddrüsenerkrankung (AITD), Typ‐1‐Diabetes mellitus und multipler Sklerose (MS).^7^, ^11^

Es wird angenommen, dass bei genetisch prädisponierten Personen Auslösefaktoren (wie Traumata, Infektionen und Medikamente) eine Hochregulierung von Adhäsionsmolekülen bewirken und eine T‐Zell‐Aktivierung auslösen. In der frühen inflammatorischen Phase sind T‐Helferzellen (Th1 und Th17) sowie die zugehörigen Zytokine vermehrt nachweisbar, während in späteren Stadien der Morphea eine Th2‐dominierte Immunantwort überwiegt.^12^, ^13^, ^14^ Zu den zentralen Zytokinen zählen *transforming growth factor β* (TGF‐β), *platelet‐derived growth factor* (PDGF) und *connective tissue growth factor* (CTGF). Diese Zytokine und Wachstumsfaktoren fördern die Aktivierung und Proliferation von Fibroblasten und führen zu einer vermehrten Ablagerung von Kollagen und extrazellulärer Matrix (ECM).^1^

### Diagnostik

#### Histopathologie

Für eine vollständige histologische Beurteilung sollte die Gewebeprobe die Subkutis einschließen.^8^, ^16^ Das zentrale histopathologische Merkmal ist die ausgeprägte Anreicherung extrazellulärer Matrix mit vermehrter Ablagerung von Kollagenfasern, Fibronectin und Elastin. In der aktiven Entzündungsphase sind verdickte Kollagenbündel parallel zur Hautoberfläche sowie entzündliche Infiltrate zu beobachten. Zum entzündlichen Infiltrat zählen Lymphozyten, Eosinophile, Plasmazellen und Histiozyten, die innerhalb der Kollagenbündel, perivaskulär sowie an der Peripherie der Hautanhangsgebilde lokalisiert sind.^7^ In späteren Stadien, wenn die Entzündung abklingt, wird die Läsion avaskulär mit verdickten Blutgefäßwänden und stark verdichteten Kollagenbündeln.

Für eine vollständige histologische Beurteilung sollte die Gewebeprobe die Subkutis einschließen. Das zentrale histopathologische Merkmal ist die ausgeprägte Anreicherung extrazellulärer Matrix mit vermehrter Ablagerung von Kollagenfasern, Fibronectin und Elastin.

Die tiefe Morphea betrifft in erster Linie das tiefe Bindegewebe mit deutlicher Sklerose und Hyalinisierung, die bis in die darunter liegenden Faszien, das Fettgewebe oder die darunter liegenden Muskeln reichen.^4^, ^17^ Bei der pansklerotischen Morphea zeigen sich in Biopsieproben Lymphozyten, Plasmazellen und Pansklerose in der gesamten Dermis und im Panniculus.^18^, ^19^

Bei der eosinophilen Fasziitis betreffen Entzündung und Fibrose vor allem die Faszien und die untere Subkutis, was zu einer Faszienverdickung führt. In der frühen Phase der eosinophilen Fasziitis umfassen die entzündlichen Infiltrate Monozyten, Plasmazellen und Eosinophile. In späteren Stadien ist die Anzahl der Entzündungszellen geringer und es gibt nur wenige oder gar keine Eosinophilen.^4^

Obwohl histologische Veränderungen bei Morphea charakteristisch sind, ermöglichen Biopsien allein keine Unterscheidung zwischen Morphea und Hautmanifestationen bei systemischer Sklerose.

#### Laboruntersuchungen

Bei Erstdiagnose sollten die Basislaboruntersuchungen ein Blutbild mit Differenzialblutbild, Entzündungsparameter wie C‐reaktives Protein und Blutsenkungsgeschwindigkeit sowie Laktatdehydrogenase und Leber‐ und Nierenfunktionswerte umfassen. Besteht der Verdacht auf eine begleitende Myositis, sollte die Kreatinkinase bestimmt werden. Liegen Arthritis‐ oder Arthralgie‐Symptome vor, kann es sinnvoll sein, Rheumafaktoren und CCP‐Antikörper zu messen. Auch der Titer antinukleärer Antikörper (ANA) sollte bestimmt werden. Bis zu 50 % der Patienten zeigen erhöhte ANA‐Werte, insbesondere Antihiston‐Antikörper (AHA) oder Anti‐Einzelstrang‐DNA‐Antikörper, während andere Autoantikörper bei weniger als 10 % nachweisbar sind.^13^ Die Positivität der drei genannten Autoantikörper ist mit schwereren Krankheitsverläufen wie der generalisierten und der gemischten Form der Morphea assoziiert. Wenn zwei oder mehr dieser Antikörper vorliegen, steigt die Wahrscheinlichkeit einer Beteiligung der tiefen Muskulatur. Die Entscheidung über die Durchführung der genannten Laboruntersuchungen wird jedoch auf Grundlage des klinischen Erscheinungsbilds des Patienten getroffen; nicht alle Parameter sind in jedem Fall erforderlich.

#### Klinische Scores

Der einzige validierte klinische Score zur Verlaufsbeurteilung der Morphea ist das *Localized Scleroderma Assessment Tool* (LoSCAT). Es umfasst den *Localized Scleroderma Skin Severity Index* (LoSSI), der die Krankheitsaktivität an 18 anatomischen Regionen auf einer Skala von 0 bis 3 erfasst, sowie den *Localized Scleroderma Skin Damage Index* (LoSDI), der das Ausmaß der krankheitsbedingten Hautschädigung anhand derselben Skala und Verteilung bewertet. Ergänzend beinhaltet das Instrument das *Physician's Global Assessment of disease activity* (PGA‐A) und *damage* (PGA‐D), die jeweils mittels 100‐mm‐visueller Analogskalen (0–100) bestimmt werden.

Weitere klinische Scores wurden vorgeschlagen, sind jedoch nicht für die Morphea validiert. Dazu zählen der *modified Rodnan Skin Score* (mRSS), der *DIET Score* (zur Beurteilung von Dyspigmentierung, Induration, Erythem und Teleangiektasien) sowie die *visual analog scale* (VAS). In jüngerer Zeit wurden zudem *Morphea Activity Measure* (MAM) und *Total Morbidity Score* (TMS) eingeführt.^20^, ^21^

#### Instrumentelle Diagnostik

Die Magnetresonanztomographie (MRT) zeigt klinisch verborgene neurologische und muskuloskelettale Beteiligungen. Eine MRT wird bei der En‐Coup‐de‐Sabre‐Variante und der progressiven fazialen Hemiatrophie nachdrücklich empfohlen, um eine Beteiligung des zentralen Nervensystems auszuschließen.^5^ Auch bei tiefer Morphea oder eosinophiler Fasziitis mit Beteiligung tiefer Gewebeschichten ist in der Regel eine MRT angezeigt.^5^

Zur Überwachung der Krankheit können neben dem 20‐MHz‐Ultraschall auch andere Verfahren wie die konfokale Reflexionsmikroskopie (RCM), die optische Kohärenztomografie (OCT), die Infrarot‐Thermografie (IRT), die Laser‐Doppler‐Durchflussmessung (LDF), das Durometer und das Cutometer in Betracht gezogen werden.^5^, ^22^, ^23^ Diese Techniken wurden in erster Linie zur Bewertung des Therapieverlaufs in klinischen Studien eingesetzt.

### Differenzialdiagnose

Bei Morphea‐Patienten ist eine gründliche Anamnese und Untersuchung auf andere Autoimmunerkrankungen angezeigt. Die Anogenitalregion sollte sorgfältig untersucht werden, da in verschiedenen Studien auf eine Koexistenz von Morphea und Lichen sclerosus hingewiesen wurde.^5^

Es gibt mehrere Subtypen der Morphea, die jeweils unterschiedliche Stadien aufweisen. Folglich gibt es zahlreiche potenzielle Differenzialdiagnosen, die in Betracht gezogen werden müssen. Eine wichtige Differenzialdiagnose für Morphea ist die systemische Sklerose (SSc). Morphea und SSc weisen gemeinsame histopathologische Merkmale auf, so dass die Histopathologie keine zuverlässige Methode zur Differenzierung der Krankheit darstellt.^24^ Morphea‐Patienten weisen weder das Raynaud‐Phänomen noch Sklerodaktylie oder Kapillarveränderungen im Nagelfalz auf, die bei SSc zu beobachten sind.^6^ Außerdem treten bei Morphea in der Regel keine Manifestationen an inneren Organen auf.

### Therapie

#### Topische Therapie

Topische Kortikosteroide gelten als der Eckpfeiler der topischen Behandlung. Empfohlen wird die tägliche Anwendung hochwirksamer Kortikosteroide (bis zu einem Monat) oder moderater Kortikosteroide (bis zu 3 Monaten) während der aktiven Phase der Morphea. Langzeittherapien sollten als Intervallbehandlung durchgeführt werden. Bei einzelnen Läsionen kann eine Anwendung unter Okklusion die Wirkung topischer Kortikosteroide verstärken.^5^ Intraläsionale Injektionen von Kortikosteroiden werden für *en coup de sabre* empfohlen.^5^ Die Injektion von 10–40 mg Triamcinolonacetonid, unverdünnt oder mit Lidocain im Verhältnis 1:2 bis 1:4 verdünnt, in den aktiven Rand kann zufriedenstellende Ergebnisse erzielen.^4^ Andere vorgeschlagene topische Therapien erfolgen off‐label und basieren auf begrenzter Datenlage. Topische Calcineurininhibitoren,^4^, ^25^, ^26^ Imiquimod und Calcipotriol^4^, ^27^ zeigten in kleinen Studien und Fallserien positive Effekte.^8^

#### Phototherapie

Die Behandlung mit ultraviolettem Licht (UV) hat nachweislich eine antifibrotische und entzündungshemmende Wirkung und reduziert die extrazelluläre Matrix durch die Aktivierung von Matrix‐Metalloproteinasen (MMP). Behandlungen auf UVA‐Basis (Wellenlängen 320–400 nm) sind besonders wirksam bei tiefen Läsionen, während Modalitäten auf UVB‐Basis (Wellenlängen 280–320 nm) bei oberflächlichen Läsionen von Vorteil sein können.^7^ Eine weitere Option ist Psoralen plus UVA (PUVA). Die PUVA‐Therapie kann in der frühen Entzündungsphase der Krankheit empfohlen werden. Aufgrund des erhöhten Risikos von Hautschäden und möglicher langfristiger Auswirkungen einer höheren UV‐Belastung wird PUVA jedoch im Allgemeinen nicht für Kinder empfohlen.^5^, ^28^

#### Systemische Therapie

Methotrexat (MTX) stellt die systemische Therapie der ersten Wahl bei Morphea mit schweren kutanen oder extrakutanen Manifestationen dar. Es wird empfohlen, die Behandlung über mindestens 12 Monate fortzuführen, um eine langfristige Krankheitskontrolle zu gewährleisten.^7^ In Fällen, in denen die Krankheit sehr aktiv und entzündlich ist, können systemische Kortikosteroide für einen begrenzten zusätzlich zu MTX verabreicht werden. Eine Monotherapie mit niedrig dosierten systemischen Kortikosteroiden kann nur bei eosinophiler Fasziitis empfohlen werden.^5^

Methotrexat (MTX) stellt die systemische Therapie der ersten Wahl bei Morphea mit schweren kutanen oder extrakutanen Manifestationen dar.

Bei Patienten, die MTX und Kortikosteroide nicht vertragen oder bei denen sie kontraindiziert sind, gilt Mycophenolatmofetil (Dosierung 0,5–2,0 g/Tag) als Zweitlinientherapie, insbesondere bei rezidivierenden oder schweren Fällen von Morphea.^29^ Bei stärker entzündlichen Formen von Morphea hat sich Abatacept als vielversprechende Zweitlinientherapie erwiesen. Es kann als Monotherapie oder in Kombination mit MTX, Kortikosteroiden oder Mycophenolatmofetil eingesetzt werden.^5^

#### Zukunftsperspektiven der Therapie

Die jüngere Forschung richtet den Fokus auf zielgerichtete Therapien mit einem günstigeren Nebenwirkungsprofil. Studien zeigten vielversprechende Ergebnisse mit Wirkstoffen, die gezielt Zytokine hemmen, darunter Tocilizumab und Sarilumab (IL‐6‐Rezeptor‐Antagonisten) sowie Infliximab (Anti‐TNF‐α). Auch Imatinib (Tyrosinkinasehemmer), Apremilast (Phosphodiesterase [PDE]4‐Hemmer) und Everolimus (mTOR‐Hemmer) haben sich in der Behandlung der Morphea als wirksam erwiesen.^30^, ^31^, ^32^

Laufende Studien prüfen die Wirksamkeit und Sicherheit von IL‐4/IL‐13‐Inhibitoren. In einer randomisierten, kontrollierten Phase‐IIa‐Studie (NCT04200755) wird insbesondere die Wirksamkeit subkutan verabreichter Injektionen von 300 mg Dupilumab im 14‐tägigen Intervall bei Patienten mit Morphea untersucht. Der Januskinase‐(JAK)‐Signaltransduktionsweg und der daran gekoppelte STAT‐Weg sind an der Pathogenese der Morphea beteiligt; die gezielte Hemmung dieses Signalwegs hat großes Interesse geweckt.^33^ In einzelnen Fällen zeigten die JAK‐Inhibitoren Tofacitinib (ein Hemmer von JAK1 und JAK3) und Baricitinib (ein Hemmer von JAK1 und JAK2) eine bessere Wirksamkeit als konventionelle Standardtherapien. Zudem konnte nachgewiesen werden, dass *Gain‐of‐Function*‐Varianten von STAT4 zur Entwicklung einer pansklerotischen Morphea führen können und der JAK‐Inhibitor Ruxolitinib sowohl dermatologische als auch entzündliche Symptome lindert.^34^

#### Behandlung der residualen Atrophie

Entstellungen infolge einer subkutanen Gewebeatrophie bei *en coup de sabre* und progressiver fazialer Hemiatrophie können erhebliche psychische Belastungen mit sich bringen.^35^ Zur Korrektur der resultierenden Atrophie kommen verschiedene rekonstruktive Verfahren zum Einsatz, darunter autologe Fetttransplantationen, dermale Filler (insbesondere Hyaluronsäure), chirurgische Techniken, poröse Polyethylenimplantate und azelluläre dermale Matrizes.

#### Physiotherapie

Bei Patienten mit Morphea – insbesondere bei der linearen, generalisierten und pansklerotischen Verlaufsform – wird eine physiotherapeutische Behandlung mit 1–2 Sitzungen pro Woche empfohlen. In der sklerotischen Phase können auch Massagen und manuelle Lymphdrainagen von Vorteil sein. Die Physiotherapie kann den Bewegungsumfang verbessern, wird jedoch in der aktiven Entzündungsphase nicht empfohlen.^7^

### Prognose

Patienten mit Morphea haben häufig einen schubförmig‐remittierenden Verlauf, insbesondere beim generalisierten Subtyp, was eine langfristige Nachsorge erforderlich macht.^36^ Die Prognose hängt stark vom Morphea‐Subtyp ab. Bei nahezu 50 % der Patienten mit begrenzter Morphea bildet sich die Erkrankung innerhalb von 2,5 Jahren zurück.^5^ Generalisierte, lineare und tiefe Verlaufsformen weisen hingegen eine längere Krankheitsdauer von durchschnittlich 5,5 Jahren auf.

Unter einer leitliniengerechten Therapie über 6 bis 12 Monate zeigen die meisten Patienten innerhalb der ersten 3 bis 12 Monate eine Besserung der Krankheitsaktivität. Die Sklerose bildet sich jedoch in der Regel deutlich langsamer zurück – häufig über einen Zeitraum von 2 bis 5 Jahren –, während eine Gewebeatrophie trotz Behandlungsende fortbestehen oder sogar fortschreiten kann.^36^

## SYSTEMISCHE SKLEROSE (SSc)

### Definition und Epidemiologie

Die systemische Sklerose (SSc) ist eine heterogene Autoimmunerkrankung, die die Haut und die inneren Organe mit einem Verhältnis von 3–6:1 zwischen Männern und Frauen befällt. Die Krankheit tritt in der Regel bei Personen im Alter von 30–40 Jahren auf, ohne dass eine rassische Dominanz erkennbar ist.^37^

Die Prävalenz von SSc liegt in Europa bei 7,2–33,9 und in Nordamerika bei 13,5–44,3 pro 100 000 Personen. Die jährliche Inzidenz liegt in Europa bei 0,6–2,3 und in Nordamerika bei 1,4–5,6 pro 100 000 Personen.^38^, ^39^

Bei Personen mit einer genetischen Veranlagung kann die Entwicklung von SSc durch Umweltfaktoren beeinflusst werden. Endothelveränderungen und immunologische Störungen (unter Beteiligung von T‐Lymphozyten und den damit verbundenen Zytokinen) führen zu Gefäßanomalien, Veränderungen der extrazellulären Matrixverbindungen und vermehrten Kollagenablagerungen.

### Klinisches Erscheinungsbild

**ABBILDUNG 8:**
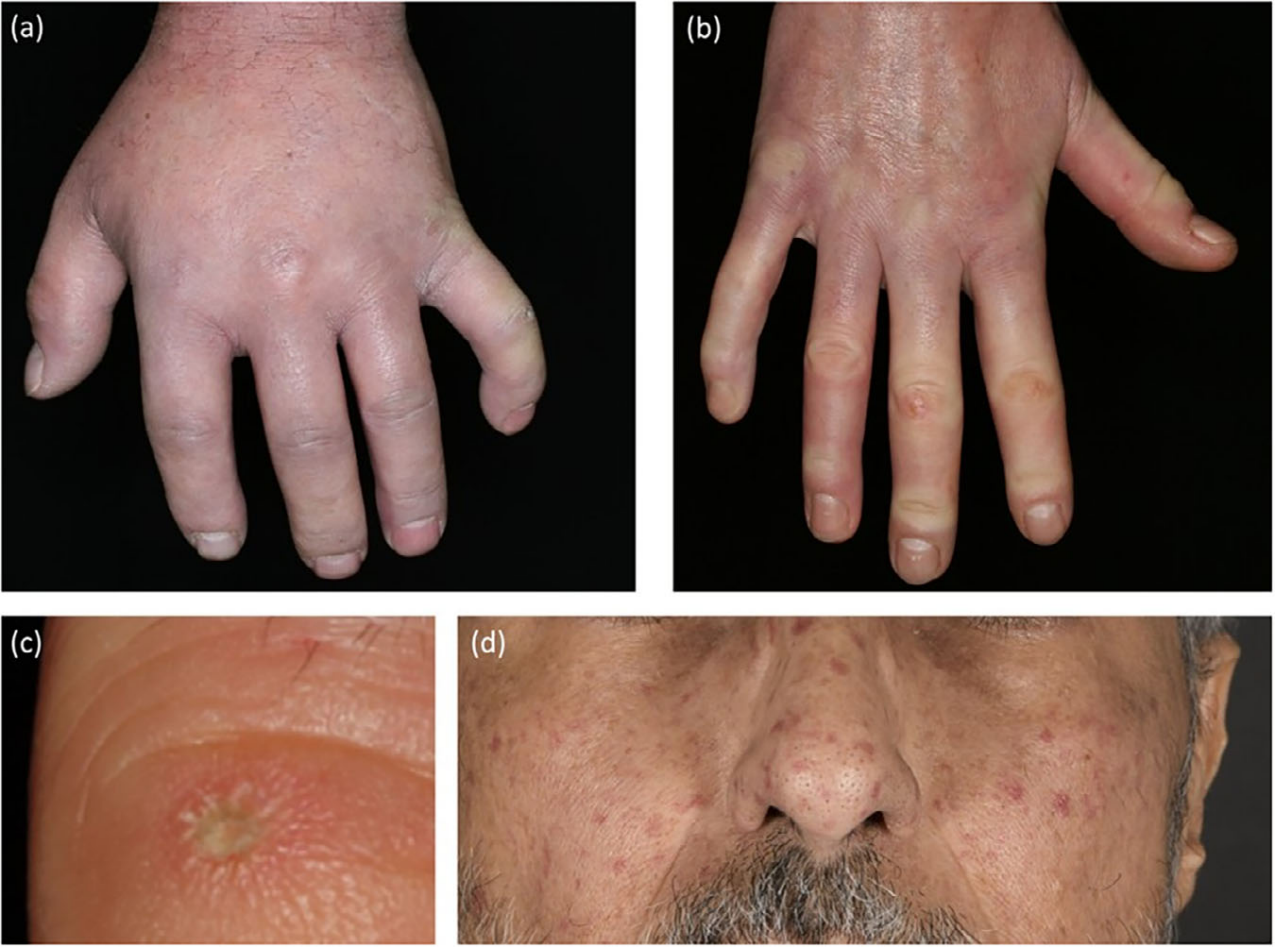
Klinisches Spektrum bei systemischer Sklerose. (a) Geschwollene Finger bei einem Patienten mit SSc; (b) Sklerodaktylie mit beginnender Kontraktur; (c) Fingerspitzennarbe mit Lochfraß; (d) Teleangiektasie im Gesicht.

Die systemische Sklerose kann sich klinisch auf unterschiedliche Weise präsentieren, indem sie die Haut und die inneren Organe befällt. Viele Patienten berichten über Müdigkeit und ein allgemeines Gefühl der Schwäche. Das Raynaud‐Syndrom tritt oft schon sehr früh im Krankheitsverlauf auf und kann anderen Symptomen manchmal um mehrere Jahre vorausgehen.^40^ Beim Raynaud‐Phänomen, das häufig durch Kälteeinwirkung oder emotionalen Stress ausgelöst wird, aber auch spontan auftreten kann, wird die Haut aufgrund einer Gefäßverengung zunächst blass. Die daraus resultierende Verminderung der Durchblutung führt zu einer lokalen Hypoxämie, die der Haut eine bläuliche Verfärbung verleiht. Auf diese Phase folgt eine reaktive Hyperämie, die schmerzhaft sein kann und zu einer Rötung sowie Erwärmung der Haut führt. Obwohl am häufigsten die Finger betroffen sind, kann das Raynaud‐Phänomen auch andere Körperregionen wie Nase, Ohren, Lippen und Mamillen betreffen.^41^ Eine weitere Frühmanifestation der Erkrankung ist die Entwicklung sogenannter „*puffy fingers*“ (Abbildung 8a), die durch Schwellung und Erythem der Finger gekennzeichnet ist.^42^ Mit dem Fortschreiten der Krankheit führt die Sklerose zu einer Verhärtung und Versteifung der Haut, was zu Sklerodaktylie führt (Abbildung 8b). Diese Versteifung schränkt die Beweglichkeit ein.^42^ In diesem Stadium ist es oft schwierig oder sogar unmöglich, die Faust vollständig zu schließen.

**ABBILDUNG 9:**
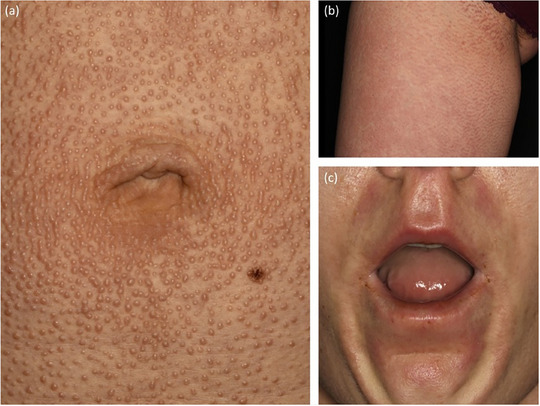
Skleromyxödem. (a, b) Ausgedehnte wachsartige Papeln; (c) peribuccale Hautverdickung.

Aufgrund wiederholter Hypoxie infolge von Raynaud‐Anfällen sowie sklerosebedingten Durchblutungsstörungen und Vaskulopathie entwickeln Patienten nicht selten Ulzerationen an den Fingern. Diese Geschwüre heilen in der Regel nur langsam ab und verursachen erhebliche Schmerzen.^43^ Zudem bilden sich im Bereich der Fingerspitzen häufig eingesunkene Narben (Lochfraß) (Abbildung 8c), und das Nagelwachstum kann beeinträchtigt sein; in manchen Fällen kommt es sogar zum vollständigen Verlust der Fingernägel.

Eine Kalzinose, definiert als Ablagerung unlöslicher Calciumsalze in der Haut und im subkutanen Gewebe, wird ebenfalls häufig an den Fingern oder an den Streckseiten der Extremitäten beobachtet.^44^ Calcinosis cutis kann auch an anderen Stellen auftreten. Während einige Fälle asymptomatisch sind, können andere zu Geschwüren führen, die starke Schmerzen und eine erheblich eingeschränkte Beweglichkeit verursachen, insbesondere wenn sie sich über Gelenken entwickeln. Patienten mit SSc weisen häufig eine sklerosebedingte Einschränkung der Gesichtsmimik auf, was allgemein als „maskenhaftes“ Aussehen beschrieben wird. Ein charakteristisches Merkmal, das als „Tabaksbeutelmund“ bekannt ist, bezieht sich auf eine Mikrostomie, die von radialen Hautfalten um den Mund begleitet wird, die die Mundöffnung einschränken. Es kann auch zu einer Sklerose des Zungenbändchens kommen, was zu einem verkürzten Zungenbändchen führt. Ein weiteres auffälliges Merkmal sind ausgeprägte Teleangiektasien, die häufig im Gesicht auftreten und für die Patienten oft ein großes kosmetisches Problem darstellen (Abbildung 8c).

In Anlehnung an das Klassifizierungssystem von LeRoy et al. wurde die SSc in der Vergangenheit nach dem Ausmaß der Hautbeteiligung in begrenzte kutane systemische Sklerose (lcSSc) und diffuse kutane systemische Sklerose (dcSSc) eingeteilt.^45^ Die diffuse Form wird als schnell fortschreitende Form der SSc beschrieben, die den Rumpf, die Extremitäten und das Gesicht betrifft. Das Raynaud‐Phänomen ist ein frühes Phänomen bei diesen Patienten und tritt in der Regel innerhalb eines Jahres nach dem Auftreten der Hautveränderungen auf. Bei den Patienten besteht ein Risiko für die Entwicklung einer Lungenfibrose. Anti‐Topoisomerase‐1‐Antikörper (ATA), auch als Anti‐Scl‐70‐Antikörper bezeichnet, sind dabei häufig nachweisbar.

Bei der limitierten Form der systemischen Sklerose (lcSSc) ist die Hautbeteiligung überwiegend auf Bereiche distal der Ellbogen‐ und Kniegelenke beschränkt. Die Patienten klagen in der Regel über ein seit langem bestehendes Raynaud‐Syndrom und eine akrale Sklerose. Bei diesen Patienten sind Antizentromer‐Antikörper (ACA) meist nachweisbar. Es besteht auch ein erhöhtes Risiko für die Entwicklung einer pulmonal‐arteriellen Hypertonie. Mit der Identifizierung zahlreicher SSc‐assoziierter Antikörper, die Aufschluss über die Krankheitsprognose und die Organbeteiligung geben, gilt die traditionelle Einteilung der SSc in diffuse und begrenzte Formen heute als überholt, wird aber in der klinischen Praxis immer noch häufig verwendet. Auch der Begriff CREST‐Syndrom, der früher zur Beschreibung eines Subtyps der lcSSc (mit Kalzinose, Raynaud‐Syndrom, Ösophagusdysmotilität, Sklerodaktylie und Teleangiektasie) verwendet wurde, gilt als überholt.

Eine muskuloskelettale Beteiligung bei systemischer Sklerose (SSc) kann sich in Form von Myalgien, Arthralgien oder Arthritis äußern. Myositiden und erhöhte Muskelenzymwerte treten ebenfalls auf, insbesondere bei Patienten mit Overlap‐Syndromen.^40^

Die interstitielle Lungenerkrankung (ILD) ist eine häufige pulmonale Komplikation der SSc, insbesondere bei Patienten mit Anti‐Scl‐70‐Antikörpern.

Die interstitielle Lungenerkrankung (ILD) ist eine häufige pulmonale Komplikation der SSc, insbesondere bei Patienten mit Anti‐Scl‐70‐Antikörpern.^46^ Die Pathogenese beruht auf der Rekrutierung und Aktivierung von Fibroblasten, was zu einer überschießenden Produktion extrazellulärer Matrix führt. Diese Akkumulation ersetzt nach und nach die normale Lungenarchitektur durch fibrotisches Gewebe, was zu einer Lungenfibrose und einer erheblichen Beeinträchtigung der Lungenfunktion führt. Die interstitielle Lungenerkrankung (ILD) kann einen raschen und progredienten Verlauf nehmen und potenziell in eine respiratorische Insuffizienz münden. Frühe klinische Anzeichen umfassen einen persistierenden trockenen Husten, belastungsabhängige Dyspnoe und Zyanose.^47^

Die pulmonale Hypertonie (PH) ist ein heterogener Zustand, und mehrere Formen können mit systemischer Sklerose (SSc) assoziiert sein, darunter die pulmonale arterielle Hypertonie (PAH), die PH aufgrund einer Linksherzerkrankung und die PH im Zusammenhang mit einer interstitiellen Lungenerkrankung.^48^ Die pulmonale arterielle Hypertonie tritt häufig bei Patienten mit Antizentromer‐Antikörpern auf.^40^ Diese Form der PH ist durch einen Gefäßumbau gekennzeichnet, der zu einer fibrotischen Verdickung und fortschreitenden Verengung der Blutgefäße führt, wodurch sich der pulmonale Gefäßwiderstand erhöht. Der erhöhte Druck belastet das rechte Herz erheblich und führt häufig zu Rechtsherzversagen und in schweren Fällen zum Tod.^49^, ^50^ Patienten mit Antizentromer‐Antikörpern haben oft nur eine leichte Hautbeteiligung und scheinen eine weniger schwere Erkrankung zu haben, was dazu führen kann, dass ein Screening auf PAH übersehen wird – ein entscheidender Aspekt angesichts der Verfügbarkeit wirksamer Therapieoptionen.

Eine kardiale Beteiligung bei SSc kann auch durch Myokardfibrose oder Erregungsleitungsanomalien aufgrund einer Fibrotisierung des kardialen Erregungsleitungssystems verursacht sein.^49^ Myokarditis und Perikarditis sind ebenfalls mögliche Manifestationen, obwohl eine sekundäre Herzbeteiligung, beispielsweise durch PAH, weitaus häufiger vorkommt. Darüber hinaus erhöht die chronische Entzündung bei SSc das Risiko einer Atherosklerose, die ebenfalls zu kardialen Komplikationen beitragen kann. Daher ist eine optimale Kontrolle des Blutdrucks und des Cholesterinspiegels für diese Patienten von entscheidender Bedeutung.

Die Nierenbeteiligung bei systemischer Sklerose ist durch eine Endothelschädigung gekennzeichnet, die zu einer Proliferation der Intima und einer Verengung der kleinen zuführenden Gefäße führt. Die daraus resultierende Verringerung des renalen Blutflusses löst eine Aktivierung des Renin‐Angiotensin‐Aldosteron‐Systems (RAAS) aus. Dieser Prozess kann zu Bluthochdruck und Niereninsuffizienz führen. Eine leichte Proteinurie, eine leicht verringerte glomeruläre Filtrationsrate oder Bluthochdruck werden daher häufig bei Patienten mit SSc beobachtet.^51^ Die Patienten können jedoch auch eine schwere und lebensbedrohliche Nierenkrise entwickeln, die zweithäufigste Todesursache bei SSc. Diese tritt vor allem bei Patienten mit Anti‐RNA‐Polymerase‐III‐Antikörpern auf, wobei sich das Risiko durch eine hochdosierte Kortikosteroidtherapie noch erhöht.^51^, ^52^ Die Nierenkrise ist durch akutes Nierenversagen gekennzeichnet, das häufig mit einer malignen Hypertonie einhergeht.

Eine gastrointestinale Beteiligung ist bei SSc sehr häufig und wird durch neurale Dysfunktion, Atrophie der glatten Muskulatur, Fibrose, Vaskulopathie, gestörte Regulation der Mikrozirkulation und Veränderungen der Darmflora verursacht.^53^, ^54^ Eine Störung der Ösophagusmotilität, insbesondere im distalen Drittel, das mit glatter Muskulatur ausgekleidet ist, tritt häufig schon früh im Krankheitsverlauf auf. Die Patienten empfinden dies in der Regel als Schluckbeschwerden.^54^ Bei bis zu 85 % der Patienten mit systemischer Sklerose (SSc) trägt eine Kardiainsuffizienz zur Entwicklung einer gastroösophagealen Refluxkrankheit (GERD) bei. Darüber hinaus verlängert eine beeinträchtigte Ösophagusperistaltik die Retention von Magensäure in der Speiseröhre, was die Schwere der GERD‐Symptome weiter verschlimmert.^55^ Eine eingeschränkte Darmmotilität kann Verstopfung und Blähungen verursachen, aber auch Durchfall aufgrund einer bakteriellen Überwucherung des Dünndarms (SIBO) ist möglich. Außerdem können Teleangiektasien im Gastrointestinaltrakt zu Blutungen führen. Im Magen, insbesondere bei Patienten mit Anti‐RNA‐Polymerase‐III‐Antikörpern, kann sich eine gastrale antrale vaskuläre Ektasie (GAVE) entwickeln, eine spezifische Form der vaskulären Ektasie, die für chronische gastrointestinale Blutungen verantwortlich ist.^56^, ^57^

### Diagnostik

#### Laboruntersuchungen

Regelmäßige Basislaboruntersuchungen, einschließlich Differenzialblutbild, sind für die Verlaufskontrolle der systemischen Sklerose (SSc) essenziell. Erhöhte Akute‐Phase‐Proteine und Gammaglobulinämie sind häufige, wenn auch nicht immer vorhandene Befunde. Routinemäßige Nierenfunktionstests und Urinuntersuchungen sollten durchgeführt werden, um eine Proteinurie festzustellen. Erhöhte NT‐proBNP‐Werte können auf eine kardiale Beteiligung hindeuten, während eine schwere Anämie auf gastrointestinale Blutungen hinweisen kann, wie beispielsweise bei einer gastralen antralen vaskulären Ektasie (GAVE). Die Überwachung der Blutfettwerte, des Blutzuckerspiegels und des HbA1c‐Wertes ist ebenfalls entscheidend für die Minimierung des Atheroskleroserisikos. Die Überwachung der Blutfettwerte, des Blutzuckerspiegels und des HbA1c‐Wertes ist ebenfalls entscheidend zur Minimierung des Atheroskleroserisikos. Abhängig von der verordneten Medikation können zusätzliche Laborparameter erforderlich sein. Zum Zeitpunkt der Erstdiagnose sollte stets eine Autoantikörperdiagnostik erfolgen (Tabelle 2).

**TABELLE 2:** Nachgewiesene Antikörper bei systemischer Sklerose und zugehörige klinische Phänotypen.

Antigen	Assoziierte klinische Befunde
Zentromer	Limitierter kutaner Subtyp Digitale Ulzerationen PAH
Topoisomerase I (Scl‐70)	Diffuser kutaner Subtyp ILD
RNA‐Polymerase III (RNAP3)	Diffuser kutaner Subtyp GAVE Nierenkrise Malignome
U3‐Ribonucleoprotein (U3‐RNP) / Fibrillarin	Frühe schwere Organbeteiligung Kardiale Beteiligung Dünndarmdysmotilität
U1‐Ribonucleoprotein (U1‐RNP)	Überlappende Syndrome
U11/U12 Ribonucleoprotein	ILD
Ku	Limitierter kutaner Subtyp
PM/Scl	Myositis‐Überlappungssyndrome Arthritis
Th/To	Limitierter kutaner Subtyp ILD PAH Perikarditis
NOR 90	Limitierter kutaner Subtyp
Eukaryotic initiation factor‐2B (eIF2B)	Diffuser kutaner Subtyp ILD

*Abk*.: PAH, pulmonal‐arterielle Hypertonie; ILD, Interstitielle Lungenerkrankungen; GAVE, gastrale antrale vaskuläre Ektasie

#### Instrumentelle Diagnostik

Der *modifizierte Rodnan Skin Score* (mRSS) ist ein klinisches Instrument zur Beurteilung der Hautbeteiligung bei SSc und zur Quantifizierung des Ausmaßes der Hautverdickung. Der mRSS umfasst eine Tastuntersuchung von 17 anatomischen Regionen, wobei jede Region auf einer Skala von 0 bis 3 bewertet wird: 0 steht für normale Haut, 1 für eine leichte Verdickung, bei der der Untersucher problemlos Hautfalten zwischen zwei Fingern bilden kann, 2 steht für eine mäßige Hautverdickung, bei der das Bilden von Hautfalten erschwert ist und keine Hautfalten (Fältelung) mehr sichtbar sind und 3 für eine schwere Verdickung, bei der es nicht möglich ist, Hautfalten zwischen zwei Untersuchungsfingern zu bilden.^58^ Der Gesamtscore ergibt sich aus der Summe der Einzelwerte, wobei maximal 51 Punkte erreicht werden können. Der mRSS korreliert häufig mit der Krankheitsaktivität und dem Fortschreiten der SSc, was ihn zu einem wichtigen Parameter sowohl für klinische Studien als auch für die Patientenbehandlung macht.

Der *modifizierte Rodnan Skin Score* (mRSS) ist ein klinisches Instrument zur Beurteilung der Hautbeteiligung bei SSc und zur Quantifizierung des Ausmaßes der Hautverdickung.

Die Kapillarmikroskopie ist ein wertvolles Instrument sowohl für die Diagnose als auch für die Überwachung der Aktivität und des Fortschreitens der SSc.^59^, ^60^ Kapillaren, die parallel zur Hautoberfläche am Nagelfalz verlaufen, können mithilfe eines Videokapillarmikroskops, Stereomikroskops, USB‐Mikroskops oder Dermatoskops untersucht werden. Die Beurteilung konzentriert sich in der Regel auf den zweiten bis fünften Finger beider Hände, wobei aufgrund der dünneren Nagelhaut die besten Ergebnisse am vierten und fünften Finger der nichtdominanten Hand zu erwarten sind. Beurteilt werden Kapillardichte, Morphologie, Durchmesser und extrakapilläre Veränderungen wie Blutungen oder Ödeme. Cutolo et al. beschrieben drei charakteristische Muster im Zusammenhang mit SSc: „früh“, „aktiv“ und „spät“.^61^ Das frühe Muster ist durch Ektasien an den Spitzen der Kapillarschlingen gekennzeichnet. Das aktive Muster zeigt sich durch das Vorhandensein von Megakapillaren mit einem Durchmesser > 50 µm, Mikroblutungen und einem mäßigen Kapillarverlust. Das späte Muster ist geprägt von avaskulären Arealen infolge eines ausgeprägten Kapillarverlusts, auf den eine Neoangiogenese folgen kann, die zur Ausbildung neuer Megakapillaren führt.

Zur Beurteilung der Lungenfunktion sollten Patienten mit SSc regelmäßig Lungenfunktionstests durchführen lassen, einschließlich der Messung der Diffusionskapazität (DLCO). Wenn Anomalien festgestellt werden, insbesondere eine Abnahme der DLCO oder der forcierten Vitalkapazität (FVC), sollte eine hochauflösende Computertomographie (HRCT) des Brustkorbs durchgeführt werden. Bei Risikopatienten, wie etwa Patienten mit Anti‐Scl‐70‐Antikörpern, kann eine HRCT bereits zum Zeitpunkt der Erstdiagnose sinnvoll sein, um einen Ausgangsbefund zu erheben. Besteht der Verdacht auf PAH, sollte zur endgültigen Diagnose eine Rechtsherzkatheteruntersuchung durchgeführt werden. Bei asymptomatischen Patienten ist ein regelmäßiges echokardiographisches Screening in der Regel ausreichend.^62^ Außerdem sollte ein Elektrokardiogramm (EKG) durchgeführt werden, um Herzrhythmusstörungen auszuschließen.^62^ Besteht der Verdacht auf eine Myokarditis, sollte eine Kardio‐MRT durchgeführt werden.

Bei einer gastrointestinalen Beteiligung können diagnostische Verfahren wie Ösophagusmanometrie, Ösophagogastroduodenoskopie (EGD) und Koloskopie eingesetzt werden, um das Ausmaß der Erkrankung zu beurteilen.

#### Diagnostische Kriterien

Im Jahr 2013 haben das *American College of Rheumatology* (ACR) und die *European League Against Rheumatism* (EULAR) diagnostische Kriterien für SSc festgelegt (Tabelle 3). Eine Diagnose kann gestellt werden, wenn eine Punktzahl von ≥ 9 Punkten erreicht wird.^63^

**TABELLE 3:** Kriterien des *American College of Rheumatology/European League Against Rheumatism* für die Klassifizierung der systemischen Sklerose.

Kriterium	Unterkriterien	Gewichtung
Verdickung der Haut an den Fingern beider Hände bis zum Metacarpophalangealgelenk		9
Hautverdickungen der Finger (es zählt nur der höhere Wert)	*Puffy fingers*	2
	Sklerodaktylie der Finger (distal der Metacarpophalangealgelenk aber proximal zu den Interphalangealgelenken)	4
Läsionen der Fingerspitzen (es zählt nur der höhere Wert)	Digitale Spitzenulzerationen	2
	*Pitting scars* (Lochfraß) der Fingerspitze	3
Teleangiektasien		2
Veränderte Kapillaren im Nagelfalz		2
Pulmonale arterielle Hypertonie und/oder interstitielle Lungenerkrankung (maximale Punktzahl: 2)	Pulmonale arterielle Hypertonie	2
	interstitielle Lungenerkrankung	2
Raynaud‐Phänomen		3
SSc‐assoziierte Autoantikörper (maximale Punktzahl ist 3)	Antizentromer Anti‐Topoisomerase I Anti‐RNA‐Polymerase III	3

In den letzten Jahren wurden die VEDOSS‐Kriterien (*Very Early Diagnosis of Systemic Sclerosis*) entwickelt, um eine möglichst frühzeitige Diagnose von SSc zu ermöglichen, noch bevor die Krankheit deutlich fortgeschritten ist.^64^ Diese Kriterien basieren auf der Erkenntnis, dass eine frühzeitige Diagnose und Behandlung das Fortschreiten der Erkrankung verlangsamen und die Prognose der Patienten verbessern können. Die VEDOSS‐Kriterien umfassen das Vorliegen eines Raynaud‐Phänomens, SSc‐spezifischer Autoantikörper und kapillarmikroskopischer Auffälligkeiten. Sie dienen der Identifikation von Personen in sehr frühen, noch nicht klassifizierbaren Stadien der Sklerodermie – bevor eine ausgeprägte Haut‐ oder Organbeteiligung auftritt.

#### Differenzialdiagnose

Die Diagnose der SSc erfolgt in erster Linie klinisch. Es müssen jedoch zahlreiche Differenzialdiagnosen in Betracht gezogen werden.⁶⁵ Dazu zählen eosinophile Fasziitis, Graft‐versus‐Host‐Erkrankung (GvHD), Überlappungssyndrome mit anderen Kollagenosen, Skleromyxödem, Sklerödem adultorum Buschke, Amyloidose, systemische nephrogene Fibrose, Stoffwechselerkrankungen wie Phenylketonurie, Exposition gegenüber toxischen Substanzen wie Silikat sowie genetische Syndrome wie das Werner‐Syndrom.

### Therapie

Die systemische Sklerose weist ein breites Spektrum an Symptomen auf und erfordert einen multidisziplinären Behandlungsansatz. Die Therapiestrategie umfasst eine Kombination aus immunsuppressiven und vasodilatatorischen Maßnahmen, die an Schweregrad und klinisches Erscheinungsbild der Erkrankung angepasst werden.

Die systemische Sklerose (SSc) geht mit einem breiten Spektrum an Symptomen einher und erfordert einen multidisziplinären Behandlungsansatz. Die Therapiestrategie umfasst eine Kombination aus immunsuppressiven und vasodilatatorischen Maßnahmen.

Bei kutaner Fibrose ist eine allgemeine Basisversorgung entscheidend. Dazu gehören das Vermeiden von Kälte und Traumata, Nikotinverzicht, die Anwendung feuchtigkeitsspendender Cremes, Lymphdrainage und Physiotherapie. Eine milde Fibrose kann mittels Phototherapie (UVA1 oder PUVA) behandelt werden, während bei progredientem Verlauf eine systemische Therapie mit Methotrexat oder Mycophenolatmofetil erforderlich ist.^66^

Bei der Behandlung des Raynaud‐Phänomens sollten Kalziumkanalblocker vom Dihydropyridin‐Typ, wie zum Beispiel orales Nifedipin, als Therapie der ersten Wahl in Betracht gezogen werden.^67^ Auch Phosphodiesterase (PDE)‐5‐Hemmer können in Betracht gezogen werden. Bei akuten Raynaud‐Anfällen ist intravenöses Iloprost eine Option, wenn orale Therapien unzureichend sind.^67^

Bei digitalen Ulzera wird zu intravenösem Iloprost oder PDE‐5‐Hemmern geraten. Bosentan sollte in Erwägung gezogen werden, um die Bildung neuer Ulzerationen zu reduzieren – insbesondere bei Patienten, die trotz Behandlung mit Kalziumkanalblockern, PDE‐5‐Inhibitoren oder Iloprost multiple Ulzera entwickeln.^67^

Patienten mit Lungenbeteiligung bei SSc sollten rasch mit immunsuppressiven Medikamenten behandelt werden. Große klinische Studien wie SLS‐I^68^ und SLS‐II^69^ haben Cyclophosphamid und Mycophenolatmofetil als krankheitsmodifizierende Therapien etabliert, die signifikante Verbesserungen der forcierten Vitalkapazität (FVC) und der radiologischen Fibrose zeigen.^70^ Seit 2020 ist auch Nintedanib, ein Tyrosinkinase‐Inhibitor, der VEGF‐, FGF‐ und PDGF‐Rezeptoren blockiert, für die Behandlung der SSc‐assoziierten interstitiellen Lungenerkrankung zugelassen.^70^ Die autologe hämatopoetische Stammzelltransplantation kann eine Option für Patienten mit progressiver diffuser kutaner SSc mit ILD sein, die nicht auf eine immunsuppressive Therapie ansprechen.

Zur Behandlung der PAH bei Patienten mit SSc werden PDE5‐Hemmer und Endothelin‐Rezeptor‐Antagonisten empfohlen, häufig in Kombination. Auch intravenöses Iloprost ist daher eine Option. Zu den neueren Zweitlinienoptionen gehören Selexipag und Riociguat, die einen zusätzlichen therapeutischen Nutzen bei der Behandlung der PAH bieten.^67^

Zur Behandlung gastrointestinaler Symptome sollten Protonenpumpeninhibitoren (PPI) eingesetzt werden, um die gastroösophageale Refluxkrankheit (GERD) zu behandeln und Speiseröhrengeschwüre zu verhindern.^67^ Obwohl es keine randomisierten kontrollierten Studien gibt, raten Experten zum Einsatz von Prokinetika, um Motilitätsstörungen wie Dysphagie, frühzeitiges Sättigungsgefühl, Blähungen und Pseudoobstruktion zu behandeln.^67^ Darüber hinaus können intermittierende oder rotierende Antibiotika zur Behandlung von SIBO eingesetzt werden, da sie möglicherweise das bakterielle Gleichgewicht im Magen‐Darm‐Trakt verbessern.^67^, ^71^ Neue therapeutische Ansätze wie Anti‐CD20‐ und Anti‐IL‐6‐Inhibitoren sowie die Behandlung mit chimären Antigenrezeptor(CAR)‐T‐Zellen werden derzeit in klinischen Studien untersucht.^70^, ^72^ Dennoch bleibt die Optimierung von Blutdruck, Blutfettwerten und Blutzuckerspiegel ein zentraler Bestandteil des Managements bei Patienten mit systemischer Sklerose.

### Prognose

Die systemische Sklerose ist eine chronische Erkrankung, die eine langfristige und multidisziplinäre Nachsorge erfordert. Alle Patienten sollten regelmäßig auf eine Organbeteiligung untersucht werden, insbesondere von Lunge, Herz, Gastrointestinaltrakt und Nieren. Eine ungünstige Prognose ist assoziiert mit höherem Lebensalter (≥ 60 Jahre), dem dcSSc‐Subtyp, Sklerodermie‐Nierenkrise, ausgeprägter Dyspnoe, eingeschränkter Lungenfunktion (FVC und DLCO [< 70 %]), Anämie sowie erhöhtem C‐reaktivem Protein (CRP > 8 mg/l).^73^

## SKLEROMYXÖDEM

### Definition und Epidemiologie

Das Skleromyxödem – auch bekannt als diffuser oder generalisierter Lichen myxoedematosus, sklerodermiformer Lichen myxoedematosus, Arndt‐Gottron‐Syndrom oder papulöse Muzinose – ist eine seltene primäre kutane Muzinose, die durch sklerotische Hautveränderungen und eine Ablagerung von Muzin in der Dermis gekennzeichnet ist und meist mit einer monoklonalen Gammopathie assoziiert ist.^74^, ^75^ Muzin ist eine Mischung aus sauren Glykosaminoglykanen, die von Fibroblasten gebildet wird und Teil der ECM ist. Muzin besitzt eine hohe Wasserbindungskapazität; bei vermehrter Muzinablagerung kommt es daher zu einem Ödem des dermalen Bindegewebes. Das Skleromyxödem unterscheidet sich von anderen Varianten des Lichen myxoedematosus, die auf die Haut begrenzt sind, da es unbehandelt einen potenziell lebensbedrohlichen Verlauf nehmen kann. Die Krankheit betrifft in der Regel Personen mittleren Alters (Durchschnittsalter 59 Jahre), wobei weder Geschlecht noch Ethnie eine Rolle spielen.^76^

Skleromyxödem – auch bekannt als diffuser oder generalisierter Lichen myxoedematosus, sklerodermiformer Lichen myxoedematosus, Arndt‐Gottron‐Syndrom oder papulöse Muzinose – ist eine seltene primäre kutane Muzinose, die durch sklerotische Hautveränderungen und eine Ablagerung von Muzin in der Dermis gekennzeichnet ist und meist mit einer monoklonalen Gammopathie assoziiert ist.

### Ätiologie und Pathogenese

Die solideste Datenlage stützt die Theorie, dass Zytokine wie Interleukin (IL)‐1, Tumornekrosefaktor (TNF)‐α und *transforming growth factor* (TGF)‐β die Glykosaminoglykansynthese sowie die Proliferation von Fibroblasten auslösen und maßgeblich zur Pathogenese des Skleromyxödems beitragen. Jüngere Untersuchungen deuten darauf hin, dass eine chronische, Th2‐dominierte T‐Zell‐Reaktion gegen ein bislang unbekanntes Zielantigen mit einer verstärkten Sekretion von IL‐4 einhergeht.^77^

### Klinisches Erscheinungsbild (Abbildung 9)

Das Skleromyxödem kann sich mit kutanen und extrakutanen Manifestationen präsentieren (Tabelle 4). Typisch für die Haut sind weitverbreitete, wachsartige Papeln mit einem Durchmesser von 2–3 mm, die bevorzugt an Kopf, Hals, Rumpf und Extremitäten auftreten. Im Verlauf der Erkrankung können sich erythematöse und indurierte Plaques entwickeln, die konfluieren und mit Hautverhärtung, Sklerodaktylie sowie einer eingeschränkten Beweglichkeit von Mund und Gelenken einhergehen.^76^, ^78^, ^79^

**TABELLE 4:** Kutane und extrakutane Merkmale des Skleromyxödems.

Organ	Klinische Merkmale
Haut	‐ Ausgedehnte, feste, wachsartige und linear angeordnete Papeln (kuppelförmig oder mit flacher Spitze) an Kopf und Hals, oberem Rumpf, Extremitäten und Oberschenkeln, unter Aussparung der Schleimhäute. ‐ Die umgebende Haut ist glänzend und verhärtet (Sklerodermoid). ‐ Vorhandensein von Erythemen, Ödemen und bräunlicher Verfärbung ‐ Die Beteiligung der Glabella ergibt löwenähnlichen Gesichtsform (leonine facies) ‐ Tiefe Furchen und tiefe Hautfalten (Shar‐Pei‐Zeichen) ‐ Zentrale, von dicker Haut umgebene Vertiefung an den Interphalangealgelenken (Doughnut‐Zeichen) ‐ Juckreiz
Nervensystem	‐ Beteiligung des peripheren Nervensystems einschließlich Karpaltunnelsyndrom oder peripherer sensorischer und motorischer Neuropathie ‐ Beteiligung des zentralen Nervensystems, einschließlich Gedächtnisverlust, Schwindel, Gangstörungen, Schlaganfall, Krampfanfälle, Psychose oder *dermato‐neuro syndrome* ‐ Augenbeteiligung, einschließlich Hornhauttrübungen und Ektropium, kann selten vorkommen
Gelenke	‐ Arthralgien ‐ Arthritis der peripheren Gelenke mit nichtentzündlichen Synovialflüssigkeiten ‐ Fibromyalgie und ausgeprägte Schwäche ‐ In seltenen Fällen Dermatomyositis, Rhabdomyolyse
Herz	‐ Kongestive Herzinsuffizienz ‐ Myokardiale Ischämie ‐ Herzblock ‐ Perikarderguss
Gastrointestinales System	‐ Dysphagie ‐ nasale Regurgitation
Lunge	‐ Dyspnoe ‐ Heiserkeit ‐ Aspirationspneumonie
Nieren	‐ Sklerodermie‐assoziierte renale Krise

Patienten mit Skleromyxödem weisen häufig eine monoklonale IgG‐Gammopathie auf, typischerweise mit Lambda‐Leichtketten. Eine Beteiligung innerer Organe ist möglich, einschließlich der Skelettmuskulatur. Betroffene können muskuläre, neurologische, rheumatologische, pulmonale, renale und kardiovaskuläre Komplikationen entwickeln.

Zu den klinischen Manifestationen können eine ösophageale Dysphagie, Muskelschwäche infolge einer Myositis, eine Enzephalopathie des zentralen Nervensystems, periphere Neuropathie, Arthropathien, eine restriktive oder obstruktive Lungenerkrankung sowie eine sklerodermieähnliche Nierenbeteiligung zählen, die häufig gemeinsam mit kutanen Symptomen auftreten.

Insbesondere die Enzephalopathie kann einen lebensbedrohlichen Verlauf nehmen. Dieses Syndrom beginnt typischerweise abrupt mit einer Verschlechterung der Hautläsionen, einem grippeähnlichen Prodrom, Fieber und Krampfanfällen und kann in ein Koma unklarer Genese übergehen.

### Diagnostik

#### Laboruntersuchungen

Empfohlene Laboruntersuchungen umfassen ein vollständiges Blutbild, ein metabolisches Panel, die Bestimmung der Muskelenzymwerte sowie eine Urinuntersuchung. Zum Nachweis einer monoklonalen Gammopathie – meist vom IgG‐Lambda‐Typ – sollten eine Serumelektrophorese und eine Immunfixation durchgeführt werden. Darüber hinaus sind Schilddrüsenfunktionsparameter erforderlich, um ein Myxödem infolge einer Schilddrüsenerkrankung auszuschließen.^80^

#### Instrumentelle Diagnostik

Zur Beurteilung der Haut kann eine hochauflösende kutane Sonographie zur Messung der Hautdicke eingesetzt werden. In der Dermatoskopie zeigen sich reiskornartige Strukturen, die den Papeln entsprechen. Die reflektierende konfokale Mikroskopie zeigt sternförmige dermale Zellen, helle Fasern und dunkle Areale, die mit den histopathologischen Befunden korrelieren.

Im Hinblick auf extrakutane Manifestationen können Tests wie die Ösophagusmanometrie (bei gastrointestinaler Beteiligung) und Lungenfunktionsuntersuchungen (bei pulmonaler Beteiligung) angezeigt sein.^80^

#### Histopathologie

Die histopathologische Beurteilung gilt als Goldstandard für die Diagnose. Die Diagnose eines Skleromyxödems basiert auf einer Trias aus *(1)* diffuser Ablagerung von Muzin (hauptsächlich Hyaluronsäure) in der oberen und mittleren retikulären Dermis, *(2)* vermehrter Kollagenablagerung und *(3)* unregelmäßig angeordnete Fibroblasten Ein interstitielles, Granuloma‐annulare‐ähnliches Muster wurde in etwa 25 % der Biopsien beschrieben.^80^, ^81^

#### Differenzialdiagnose

Es müssen verschiedene Differenzialdiagnosen berücksichtigt werden. Zu den wichtigsten zählen die systemische Sklerose, das Sklerödem, das generalisierte Myxödem im Rahmen einer schweren Hypothyreose sowie die nephrogene systemische Fibrose. Darüber hinaus sollten auch andere Erkrankungen mit sklerodermiformen Hautveränderungen in die Differenzialdiagnose einbezogen werden. Die Diagnose des Skleromyxödems wird gestützt durch das Vorliegen linearer papulöser Effloreszenzen, den Nachweis einer monoklonalen IgG‐Gammopathie sowie die charakteristische histopathologische Trias.^80^

### Therapie

Angesichts der vielfältigen kutanen und extrakutanen Manifestationen ist ein multidisziplinärer Behandlungsansatz unter Einbeziehung von Rheumatologen, Gastroenterologen und Neurologen unerlässlich.^82^ Das Skleromyxödem weist einen unvorhersehbaren, häufig progredienten Verlauf auf, was eine rasche Einleitung der Therapie und eine regelmäßige Verlaufskontrolle erforderlich macht. In der Regel ist eine langfristige Erhaltungstherapie notwendig, da es nach Absetzen der Behandlung häufig zu Rückfällen kommt. Intravenös verabreichte Immunglobuline (IVIG) gelten als bevorzugte Erstlinientherapie.^83^, ^84^ Nach ein bis zwei IVIG‐Zyklen lässt sich oftmals eine deutliche Besserung der kutanen und extrakutanen Symptome beobachten, insbesondere im rheumatologischen Bereich. Bei unzureichendem Ansprechen auf die Initialtherapie oder bei schwerer Verlaufsform können zusätzliche Behandlungsoptionen in Betracht gezogen werden, darunter Glukokortikoide (zum Beispiel Prednison, Dexamethason) oder andere immunmodulierende Substanzen wie Thalidomid und Lenalidomid. Auch der gegen CD38 gerichtete monoklonale Antikörper Daratumumab zeigte in einer begrenzten Anzahl von Studien Wirksamkeit.^85^, ^86^, ^87^, ^88^

## SCLEROEDEMA ADULTORUM BUSCHKE

Das Skleroedema adultorum Buschke (Sklerödem) ist eine seltene sklerosierende Hauterkrankung, bei der mehr als die Hälfte der Betroffenen jünger als 20 Jahre ist. Obwohl genaue Prävalenz‐ und Inzidenzdaten fehlen, weisen Studien auf eine Prävalenz von 2,5 % bis 14,0 % bei Patienten mit Diabetes mellitus hin. Ein geschlechtsspezifisches oder ethnisches Verteilungsmuster wurde nicht beobachtet.

Das Sklerödem kann in drei Typen unterteilt werden: *(1)* Sklerödem nach Infektionen, *(2)* Sklerödem im Zusammenhang mit hämatologischen Erkrankungen und monoklonaler Gammopathie, *(3)* Sklerödem bei Diabetes mellitus.

Das Sklerödem tritt hauptsächlich im Zusammenhang mit drei klinischen Konstellationen auf und lässt sich entsprechend klassifizieren (Tabelle 5): *(1)* Sklerödem nach Infektionen, am häufigsten nach einer Streptokokkenpharyngitis und häufig begleitet von Fieber; *(2)* Sklerödem im Zusammenhang mit hämatologischen Erkrankungen und monoklonaler Gammopathie, wie zum Beispiel beim multiplen Myelom; und *(3)* Sklerödem bei Diabetes mellitus (Skleroedema diabeticorum).^91^

**TABELLE 5:** Klinische Kernpunkte des Skleroedema adultorum Buschke.

Sklerödem	Grunderkrankungen	Krankheitsverlauf	Therapie	Prognose
Typ 1	Infektion, (Streptokokken‐ oder Virusinfektion der Atemwege)	Plötzliches Auftreten mit Fieber	Selbstlimitierend (*watch and wait*) Phototherapie (UVA1 oder Psoralen mit UVA [PUVA]) Bei entsprechender Indikation Antibiotika	Positiv (Remission innerhalb von 24 Monaten)
Typ 2	Hämatologische Erkrankungen, Paraproteinämie, monoklonale Gammopathie, multiples Myelom, Amyloidose	Schleichender Beginn, langsam fortschreitend mit chronischem Verlauf	Behandlung der Grunderkrankung	Schlechte Prognose bei persistierenden Läsionen Mögliche systemische Beteiligung mit hoher Morbidität und Mortalität
Typ 3	Diabetes mellitus	Schleichender Beginn, langsam fortschreitend mit chronischem Verlauf	Behandlung der Grunderkrankung	Schlechte PrognoseChronisch progressiver Verlauf Systemische Beteiligung

### Pathogenese

Die genaue Pathophysiologie des Sklerödems ist bislang nicht bekannt. Es wird angenommen, dass verschiedene Auslöser wie Infektionen (zum Beispiel Streptokokken), mikrovaskuläre Schädigungen, Hypoxie, Medikamente sowie genetische Prädispositionen zu einer übermäßigen Produktion von Muzin und Kollagen durch Fibroblasten führen.^92^ Beim diabetesassoziierten Sklerödem könnte die irreversible Glykosylierung von Kollagen sowie eine veränderte Kollagenaseaktivität eine Rolle spielen.^80^

### Klinisches Erscheinungsbild

**ABBILDUNG 10:**
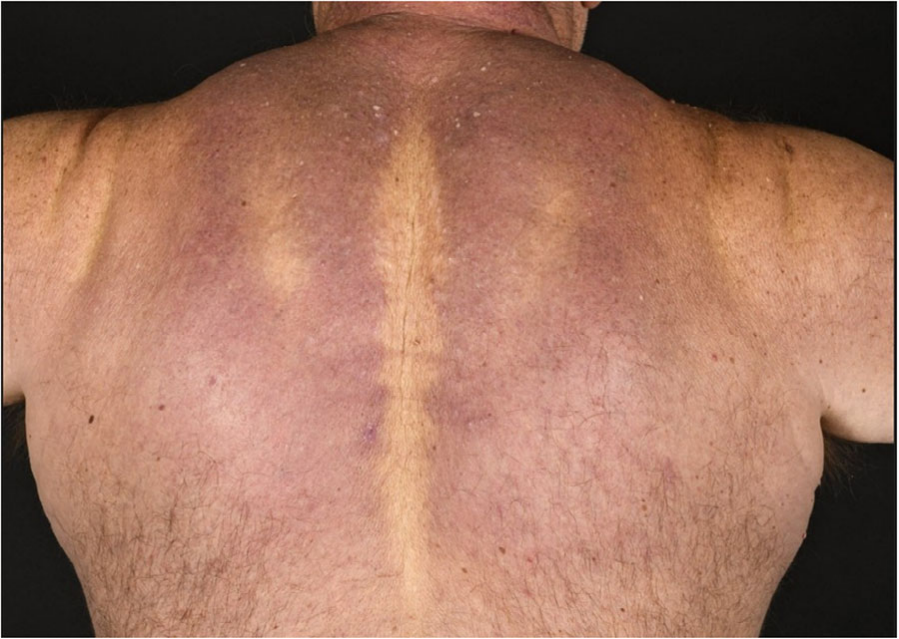
Hautverdickung im Bereich des Halses und des oberen Rückens bei einem Patienten mit Scleroedema adultorum Buschke.

Je nach Typ des Sklerödems kann das klinische Bild sowohl kutane als auch extrakutane Manifestationen umfassen. Die Hautveränderungen, die allen drei Formen gemeinsam sind, beginnen typischerweise mit einer ausgedehnten, diffusen, holzartigen Induration der Haut, die häufig von einem Erythem und einem orangenhautartigen Aspekt (peau‐d'orange) begleitet wird. Die Hautverdickung beginnt in der Regel am Hals oder oberen Rumpf und betrifft charakteristischerweise nicht die akralen Areale (Abbildung 10). In schweren Fällen können Hautversteifungen zu eingeschränkter Beweglichkeit und funktionellen Beeinträchtigungen führen. Eine extrakutane Beteiligung kann das gastrointestinale, muskuloskelettale, okuläre, respiratorische oder kardiovaskuläre System betreffen.^93^

### Diagnostik

#### Laboruntersuchungen

Der Antistreptolysin‐O‐(ASO)‐Titer kann zur Erfassung einer kürzlich zurückliegenden Streptokokkeninfektion bestimmt werden. Zur Abklärung eines Diabetes mellitus sollten der Nüchternblutzucker oder der HbA1c‐Wert erhoben werden. Zur Beurteilung möglicher monoklonaler Gammopathien werden ein vollständiges Blutbild, ein Differenzialblutbild, eine Serumelektrophorese und eine Immunfixation empfohlen. Auffällig ist, dass antinukleäre Antikörper (ANA) beim Sklerödem in der Regel negativ sind.^80^

#### Instrumentelle Diagnostik

Zur Beurteilung der Hautverhärtung können Geräte wie ein Durometer oder die Ultraschalluntersuchung eingesetzt werden.^94^ Abhängig vom Schweregrad der Erkrankung und einer möglichen systemischen Beteiligung können weiterführende Untersuchungen wie Lungenfunktionstests (bei pulmonaler Beteiligung) oder eine ösophageale Manometrie (bei gastrointestinaler Beteiligung) angezeigt sein.^80^

#### Klinische Scores

Angesichts der Seltenheit des Sklerödems existiert bislang kein spezifischer klinischer Score für diese Erkrankung. Der mRSS kann jedoch zur Einschätzung des Ausmaßes der Hautbeteiligung herangezogen werden.^95^

#### Histopathologie

Die Epidermis ist in der Regel intakt und unauffällig. Die Dermis ist aufgrund vergrößerter Kollagenfaserbündel und dazwischenliegender, mit Muzin (meist Hyaluronsäure) gefüllter Areale drei‐ bis viermal dicker als gewöhnlich. Erwähnenswert ist, dass das Fehlen von Muzin den Befund eines Sklerödems nicht ausschließt. Eine Proliferation von Fibroblasten liegt beim Sklerödem nicht vor. Die elastischen Fasern können zahlenmäßig reduziert sein.

### Differenzialdiagnose

Das Sklerödem kann klinische Merkmale aufweisen, die denen anderer sklerosierender Erkrankungen ähneln. Zu den wichtigsten Differenzialdiagnosen zählen die systemische Sklerose, das Skleromyxödem, das Myxödem, die eosinophile Fasziitis, die kutane Amyloidose, das Lymphödem sowie die *Graft‐versus‐Host*‐Erkrankung.^80^

#### Therapie

Bei allen Typen des Sklerödems ist eine physikalische Therapie angezeigt, um funktionellen Einschränkungen entgegenzuwirken. Bei ausgeprägter Hautverhärtung oder systemischer Beteiligung stellt die Phototherapie (PUVA, UVA1 oder Schmalband‐UVB) die Erstlinientherapie dar; in zweiter Linie kann eine systemische Behandlung mit Methotrexat (MTX) oder Glukokortikoiden erfolgen.^96^ Da die Streptokokken‐assoziierte Form in der Regel selbstlimitierend verläuft, ist eine systemische Therapie hier nur selten erforderlich.

### Prognose

Patienten mit Sklerödem sollten regelmäßig nachverfolgt und gezielt auf Paraproteinämien sowie systemische Komplikationen untersucht werden.^97^ Beim Sklerödem beschränken sich die Symptome in der Regel auf die Haut, in Einzelfällen kann jedoch auch eine systemische Beteiligung auftreten. Die Gesamtprognose hängt vom jeweiligen Subtyp des Sklerödems ab. Die günstigsten Verläufe zeigen sich bei postinfektiösen Formen, während Sklerödeme im Zusammenhang mit hämatologischen Erkrankungen oder Diabetes mellitus in der Regel mit einer ungünstigeren Prognose einhergehen.

## SELTENE SKLEROSIERENDE HAUTERKRANKUNGEN

### Nephrogene systemische Fibrose

Die nephrogene systemische Fibrose (NSF) ist eine therapieresistente Erkrankung, die klinisch durch indurierte Plaques gekennzeichnet ist, die symmetrisch an den Extremitäten und am Rumpf auftreten. In schweren Fällen wurde auch eine Beteiligung innerer Organe wie Herz, Skelettmuskulatur und Lunge beschrieben.^98^ Die Erkrankung wurde Anfang der 2000er‐Jahre erstmals beschrieben und seitdem mit der Anwendung gadoliniumhaltiger Kontrastmittel in Verbindung gebracht. Besonders gefährdet sind Patienten mit terminaler Niereninsuffizienz, bei denen sich nach der Gabe von Gadolinium eine NSF entwickeln kann.^99^ Aus diesem Grund ist der Einsatz bestimmter gadoliniumhaltiger Kontrastmittel – insbesondere Gadopentetat‐Dimeglumin (Magnevist^®^), Gadodiamid (Omniscan™) und Gadoversetamid (OptiMARK™) – bei dieser Patientengruppe strikt zu vermeiden.

### Toxisches Ölsyndrom

Das toxische Ölsyndrom wurde durch die Aufnahme von mit Anilin verunreinigtem, aufbereitetem Rapsöl verursacht und trat in den 1980er‐Jahren in Spanien auf. Betroffene zeigten initial makulopapulöse Hautveränderungen in Kombination mit grippeähnlichen Symptomen. Im weiteren Verlauf erfasste der Entzündungsprozess auch das Nervensystem, die Lunge und die Speicheldrüsen. Die Hautmanifestationen entwickelten sich von exanthematischen Läsionen zu sklerotischen, Morphea‐ähnlichen Veränderungen.^100^

### Eosinophilie‐Myalgie‐Syndrom

Das Eosinophilie‐Myalgie‐Syndrom trat 1989 in den Vereinigten Staaten auf und wurde mit einer verunreinigten L‐Tryptophan‐Präparation in Verbindung gebracht, die von vielen Betroffenen als Nahrungsergänzungsmittel eingenommen worden war. Die Erkrankung äußerte sich in systemischen Symptomen wie Myalgien, Fieber, Dyspnoe, Ödemen, peripherer Eosinophilie und erythematösen Hautveränderungen. Etwa die Hälfte der Patienten entwickelte im weiteren Verlauf eine ausgeprägte Induration der Extremitäten, begleitet von einer progredienten peripheren Neuropathie und Myopathie.^101^

### Stiff‐Skin‐Syndrom

Das *Stiff‐Skin*‐Syndrom (SSS) ist eine pädiatrische Erkrankung, die erstmals 1971 beschrieben wurde und typischerweise bereits bei Geburt oder im frühen Kindesalter auftritt.^102^ Klinisch ist sie gekennzeichnet durch eine ausgeprägte Induration der Haut und des darunterliegenden Gewebes, wobei vor allem die Oberschenkel und das Gesäß betroffen sind. Diese Veränderungen können die Beweglichkeit erheblich einschränken; eine viszerale Beteiligung liegt jedoch nicht vor. Häufig zeigen betroffene Kinder eine milde Hypertrichose. Das SSS wird durch Mutationen im *FBN1*‐Gen verursacht, das für Fibrillin‐1 kodiert. Die daraus resultierende Fehlregulation führt zu einer gesteigerten TGF‐β‐Aktivität, die zur charakteristischen Hautverhärtung beiträgt.^103^

## DANKSAGUNG

Yasamin Kalantari ist Stipendiatin im Rahmen eines von der EADV finanzierten Forschungsstipendiums.

Open access Veröffentlichung ermöglicht und organisiert durch Projekt DEAL.

## INTERESSENKONFLIKT

Keiner.

## [[CME Questions/Lernerfolgskontrolle]]

1. Welche Aussage zur Plaque‐Form der Morphea ist korrekt?
  - Sie ist die häufigste Unterform der limitierten Morphea.
  - Sie betrifft vor allem die Extremitäten.
  - Klinisch zeigt sie sich durch disseminierte münzgroße Herde.
  - Sie tritt vorwiegend bei Kindern unter 14 Jahren auf.
  - Sie zeigt fast immer eine symmetrische Verteilung an mindestens drei anatomischen Stellen.
2. Bei welchem Subtyp der Morphea ist eine Beteiligung subkutaner Strukturen ausgeschlossen?
  - Eosinophile Fasziitis
  - *Disabling pansclerotic morphea*
  - *en coup de sabre*
  - Lineare Morphea
  - Atrophodermie von Pasini Pierini
3. Welche laborchemische Untersuchung wird bei der Erstdiagnose einer Morphea **nicht** empfohlen?
  - ANA‐Titer
  - Differenzialblutbild
  - Borrelien‐Serologie
  - Laktatdehydrogenase
  - Creatinkinase
4. Welche Therapie hat bei der Behandlung der Morphea einen relevanten Stellenwert?
  - 5‐Fluorouracil
  - Methotrexat
  - Topische Immunmodulation mit Diphenylcyclopropenon
  - Spezifische Immuntherapie
  - Rituximab
5. Welche Aussage zur Prognose der Morphea trifft zu?
  - Die Erkrankung endet häufig letal.
  - Der Subtyp der Morphea hat keinen Einfluss auf die Prognose.
  - Die Plaque‐Form der Morphea verläuft besonders langwierig im Vergleich zu den anderen Subtypen.
  - Am besten kann das Therapieansprechen durch die Beurteilung der Atrophie evaluiert werden.
  - Vor allem der generalisierte Subtyp zeigt häufig langwierige chronische Verläufe.
6. Welche Untersuchung ist bei Erstdiagnose einer systemischen Sklerose **nicht** sinnvoll?
  - Lungenfunktion mit CO‐Diffussionskapazität
  - Untersuchung der Nagelpfalzkapillaren
  - Urinstatus
  - Basophilen‐Aktivierungstest
  - Erhebung des modifizierten *Rodnan Skin Scores*
7. Was ist ein typisches frühes Symptom der systemischen Sklerose?
  - Myokardinfarkt
  - Raynaud‐Phänomen
  - Gelenkdeformitäten
  - Schwere pulmonale Hypertonie
  - Zirrhose der Leber
8. Was ist ein typisches Merkmal der „späten“ Phase in der Kapillarmikroskopie bei systemischer Sklerose?
  - Vermehrte Kapillardichte
  - Torquierte Kapillaren
  - Avaskuläre Areale und Neoangiogenese
  - Keine Veränderungen im Kapillarbett
  - Hohe Anzahl kleiner, unauffälliger Kapillaren
9. Welche Untersuchung ist bei Scleroedema adultorum Buschke sinnvoll?
  - HbA1c
  - Pneumokokken‐PCR
  - Nachweis des Philadelphia‐Chromosoms
  - Quantiferontest
  - Gesamt‐IgE
10. Welche diagnostische Methode wird als Goldstandard für die Diagnose des Skleromyxödems angesehen?
  - Serum‐Elektrophorese zur Bestimmung der monoklonalen Gammopathie
  - Digitale Dermatoskopie mit Beurteilung der Nagelfalzkapillaren
  - Histopathologische Untersuchung der Hautbiopsie
  - Hochauflösender Ultraschall zur Messung der Hautdicke
  - Genetische Untersuchung mit Mutationsnachweis

**Lösungen**: 1a, 2e, 3c, 4b, 5e, 6d, 7b, 8c, 9a, 10c
